# Neoadjuvant immunochemotherapy plus thymalfasin in locally advanced gastric cancer: a prospective clinical trial

**DOI:** 10.1186/s12916-026-04740-z

**Published:** 2026-02-26

**Authors:** Hao Xu, Fengyuan Li, Bowen Li, Dinghua Yang, Tianyu Liu, Yiwen Xia, Hongjin Hua, Qiong Li, Jian Wang, Hongda Liu, Zekuan Xu

**Affiliations:** 1 Department of General Surgery, The First Affiliated Hospital of Nanjing Medical University, Nanjing, China; 2 Gastric Cancer Centre, The First Affiliated Hospital of Nanjing Medical University, Nanjing, China; 3 Department of General Surgery, Sir Run Run Hospital of Nanjing Medical University, Nanjing, China; 4 Department of Pathology, First Affiliated Hospital of Nanjing Medical University, Nanjing, China; 5 Department of Radiology, First Affiliated Hospital of Nanjing Medical University, Nanjing, China; 6 The Second People’s Hospital of Changzhou, Changzhou, China

**Keywords:** Gastric cancer, Neoadjuvant immunochemotherapy, Thymalfasin, Anti-PD-1, RNA sequencing

## Abstract

**Background:**

Neoadjuvant immunochemotherapy has emerged as a promising strategy for locally advanced gastric and gastroesophageal junction (G/EGJ) adenocarcinoma, but a substantial proportion of patients derive limited benefit. Thymalfasin is an immunomodulatory peptide that may amplify antitumor immunity while attenuating toxicity. We conducted a phase II trial to evaluate anti-PD-1 plus SOX (S-1 and oxaliplatin) immunochemotherapy combined with thymalfasin as neoadjuvant treatment for G/EGJ adenocarcinoma.

**Methods:**

The prospective trial enrolled patients aged 18–75 with cStage III G/EGJ adenocarcinoma, ECOG 0–1, and adequate organ function. Treatment included three 21-day cycles of serplulimab (anti-PD-1) plus SOX (S-1 and oxaliplatin) and 9 weeks of thymalfasin, followed by curative gastrectomy. The primary endpoint was pathological complete response (pCR). Secondary endpoints included major pathological response (MPR), safety, survival, and other efficacy measures. Peripheral immune remodeling was assessed by flow cytometry, and bulk RNA sequencing of peripheral blood mononuclear cells (PBMCs) interrogated thymalfasin-associated transcriptional programs.

**Results:**

Thirty patients were enrolled and all underwent curative-intent minimally invasive gastrectomy. pCR was achieved in 30.0% (9/30), and MPR in 56.7% (17/30). ypN0 status was observed in 63.3% (19/30), with N-stage downstaging in 80.0% (24/30). At a median follow-up of 14.0 months (range 10.0–17.2), only one retroperitoneal nodal relapse had occurred at 14.4 months after diagnosis; no deaths were documented. Any-grade adverse events (AEs) occurred in 93.3% of patients, grade ≥ 3 AEs in 26.7%, and immune-related AEs in 23.3%. Flow cytometry showed expansion of CD8⁺ T cells with increased CD69 expression and a concurrent reduction in HLA-DR-positive T cells, suggesting dynamic remodeling from broad systemic activation toward a more focused effector or memory response. RNA-seq revealed thymalfasin-associated upregulation of genes involved in antigen processing and presentation, type I interferon signaling, and identified immune co-expression modules linked to treatment and response.

**Conclusions:**

Neoadjuvant serplulimab, SOX, and thymalfasin produced encouraging pathological response, substantial nodal clearance, and an acceptable safety profile in stage III G/EGJ adenocarcinoma. Peripheral immune and transcriptomic profiling are consistent with a hypothesis in which thymalfasin may help preserve and coordinate systemic antitumor immunity without excessive toxicity. These findings warrant further larger randomized trials.

**Trial registration:**

ClinicalTrials.gov, NCT06461910, 2024–06-14.

**Supplementary Information:**

The online version contains supplementary material available at 10.1186/s12916-026-04740-z.

## Background

Gastric and gastroesophageal junction (G/EGJ) adenocarcinomas remain major global health challenges, characterized by aggressive disease progression and poor long-term prognosis [1, 2]. Surgical resection has long been the cornerstone of potentially curative treatment; however, surgery combined with postoperative adjuvant therapy often falls short in significantly improving survival outcomes, particularly in patients with locally advanced disease [3–7]. Consequently, increasing studies emphasize the importance of perioperative strategies to optimize resectability and enhance survival outcomes in advanced-stage disease. Despite these efforts, neoadjuvant chemotherapy alone has demonstrated limited effectiveness. For example, the PRODIGY study, which employed a DOS regimen (docetaxel, oxaliplatin, and S-1), achieved a pathologic complete response (pCR) rate of only 10.4%, while the RESOLVE trial reported a mere 5.6% pCR rate with preoperative SOX (oxaliplatin plus S-1) [8, 9]. These modest outcomes highlight the pressing need for more effective neoadjuvant strategies, prompting investigations into combinatory approaches such as preoperative immunochemotherapy.

Immunotherapy, particularly immune checkpoint inhibitors targeting programmed death receptor-1 (PD-1) and its ligand (PD-L1), has revolutionized cancer treatment across multiple malignancies [10]. In the context of gastric cancer, neoadjuvant immunochemotherapy has shown promising potential by enhancing antitumor immune responses and promoting tumor regression before surgical resection [11]. However, clinical benefits remain inconsistent and are often closely associated with biomarkers such as PD-L1 expression and DNA mismatch repair (MMR) status [12]. The KEYNOTE-585 trial, for example, evaluated pembrolizumab combined with chemotherapy versus chemotherapy alone in a neoadjuvant setting. While no significant difference in pCR rate was observed among patients with PD-L1 combined positive score (CPS) < 1, those with CPS ≥ 1 demonstrated a more than 12% higher pCR rate in the pembrolizumab arm [13]. Similar trends have been observed in trials evaluating immunotherapy in advanced gastric cancer. In the CheckMate 649 and KEYNOTE-859 studies, nivolumab or pembrolizumab combined with chemotherapy showed substantial survival benefits primarily in patients with higher PD-L1 expression, while those with CPS < 1 exhibited minimal improvement in median overall survival (OS) and progression-free survival (PFS) [14, 15]. These findings underscore the critical role of PD-L1 expression as a predictive biomarker for immunotherapy efficacy in gastric cancer. Nonetheless, they also highlight a substantial patient population with low PD-L1 expression that remains unresponsive to current immunotherapy strategies, driving the need for novel therapeutic combinations to broaden the clinical benefit.

Previous studies have suggested that further modulation of the immune microenvironment may enhance therapeutic responses in malignancies [16, 17]. This observation has led to investigations into novel therapeutic combinations that incorporate immune modulators to improve clinical outcomes. Among these, thymalfasin, an immunomodulatory peptide, has garnered attention for its capacity to stimulate immune responses across various clinical settings [18]. Emerging preclinical and retrospective clinical studies indicate that thymalfasin not only augments the efficacy of immune checkpoint inhibitors but may also mitigate treatment-associated adverse events [19–22]. Mechanistically, thymalfasin is known to activate dendritic cells through Toll-like receptor (TLR) pathways, thereby enhancing the activity of natural killer (NK) cells and cytotoxic T lymphocytes (CTLs) [23–25]. This immune activation is complemented by its ability to promote regulatory T cell (Treg) expansion and modulate cytokine production, contributing to a balanced immune response that enhances antitumor activity while potentially reducing immune-mediated toxicity.

Despite these promising insights, no clinical studies to date have evaluated thymalfasin in combination with neoadjuvant immunochemotherapy for locally advanced G/EGJ adenocarcinoma. To address this gap, we designed a phase 2 clinical trial (GATES) to investigate the efficacy and safety of a neoadjuvant regimen comprising serplulimab (an anti-PD-1 monoclonal antibody), thymalfasin, and SOX chemotherapy. This combination aims to leverage the immune-priming effects of thymalfasin alongside checkpoint inhibition and cytotoxic chemotherapy to enhance pathological responses and clinical downstaging. Additionally, we aimed to evaluate the safety profile of this approach, hypothesizing that thymalfasin might reduce immune-related adverse events (irAEs) commonly associated with checkpoint inhibitors. We further incorporated peripheral immune phenotyping and transcriptomic profiling to explore thymalfasin-related immunologic mechanisms.

## Methods

### Study design and participants

This was a prospective, open-label, phase II clinical trial conducted at First Affiliated Hospital of Nanjing Medical University, aiming to evaluate the efficacy and safety of neoadjuvant therapy combining serplulimab, thymalfasin, and the SOX chemotherapy regimen in patients with locally advanced G/EGJ adenocarcinoma. Eligible participants included males and females aged 18–75 years diagnosed with HER2-(-/1 +) cStage III (cT3-4aN1-3M0) adenocarcinoma, based on the AJCC 8th edition criteria and confirmed by gastroscopy, pathology, and imaging. Patients must have had no prior systemic treatment, ECOG performance status of 0–1, measurable lesions according to RECIST v1.1, normal organ function, and life expectancy ≥ 3 months. Detailed inclusion criteria encompassed adequate hematological, hepatic, renal, and coagulation parameters, and exclusion criteria included active infections, autoimmune diseases requiring systemic therapy, previous malignancies within 5 years, and conditions increasing the risk of immunotherapy (Additional file 1: Study protocol). This trial is registered with ClinicalTrials.gov, NCT06461910.

### Treatment protocol

The patients were enrolled between June 2024 and December 2024. No protocol modifications were made throughout the duration of the study. Upon enrollment, patients provided informed consent and underwent comprehensive baseline assessments, including gastroscopy, pathology tests, imaging studies (abdominal CT/MRI), cardiac evaluations, laboratory tests, and evaluations of PD-L1 expression and microsatellite stability. Following these assessments, patients commenced neoadjuvant therapy consisting of three cycles (each cycle 21 days) of intravenous serplulimab (300 mg, day 1), subcutaneous thymalfasin (4.8 mg twice weekly, day 1 and day 4 of each week), intravenous oxaliplatin (130 mg/m^2^, day 1), and oral S-1 (dose based on body surface area, administered twice daily from days 1–14).

Safety and efficacy assessments occurred periodically. Adverse events were evaluated clinically on day 1 of each subsequent cycle. Imaging evaluations to assess tumor response and surgical feasibility were conducted after cycles 2 and 3. Radical gastrectomy with D2 lymphadenectomy was performed within 2–6 weeks following completion of neoadjuvant therapy. Postoperative management was determined by clinical judgment and patient preference.

### Pathological assessment

PD-L1 expression was evaluated immunohistochemically using the 22C3 pharmDx assay (Agilent Technologies) and reported as the Combined Positive Score (CPS). All surgical specimens were processed and evaluated according to standardized institutional protocols. Pathological response in the primary tumor was graded using the AJCC/NCCN-based Tumor Regression Grade (TRG) system for gastric cancer. The grades were defined as follows: TRG 0 (pCR): no viable tumor cells; TRG 1: minimal residual tumor with single cells or small clusters; TRG 2: residual tumor less than fibrosis; TRG 3: extensive residual tumor with minimal fibrosis. For this study, a pathological complete response (pCR) was defined as TRG 0 (ypT0N0), and a major pathological response (MPR) was defined as TRG 0 or 1 (≤ 10% residual viable tumor).

Lymph nodes were assessed via meticulous dissection of all perigastric and extra-perigastric adipose tissue. All identified lymph nodes were entirely submitted for histological examination, with larger nodes sectioned to optimize detection of metastases. The post-therapy pathological nodal stage (ypN) was assigned according to the AJCC 8th edition staging manual, based on the absolute number of lymph nodes containing metastatic carcinoma.

### Outcomes

The primary outcome was the pathological complete response (pCR) rate, defined as the absence of viable tumor cells microscopically in all resected specimens. Secondary outcomes included the major pathological response (MPR, ≤ 10% residual viable tumor), downstaging rates, R0 resection rate, objective response rate (ORR), disease control rate (DCR), relapse-free survival, etc. Pathological responses were assessed by two independent pathologists blinded to the patients’ clinical information. Safety outcomes encompassed treatment-emergent adverse events (TEAEs) graded according to Common Terminology Criteria for Adverse Events (CTCAE v5.0), immune-related adverse events (irAEs), and perioperative complications classified by Dindo-Demartines-Clavien criteria. Survival and relapse were followed-up every 3 months, and the median follow-up time was 14.0 months (ranging 10.0–17.2 months).

### Peripheral blood lymphocyte subset analysis

Peripheral venous blood samples were collected from 20 patients at two time points: prior to the initiation of neoadjuvant therapy (baseline) and after completion of planned treatment cycles, but before surgical resection. The remaining 10 patients from the ITT population were not included in these analyses due to insufficient PBMC sample quality (e.g., low cell viability or yield) required for comprehensive profiling, or because additional blood sampling for translational research was declined by the patient. Samples were processed within 4 h of collection to ensure cellular integrity. Peripheral blood mononuclear cells (PBMCs) were isolated using density gradient centrifugation with BY-600c (Baiyang, Inc). Flow cytometry was employed to characterize major lymphocyte subsets and their activation status. Cell suspensions were stained with a panel including antibodies against CD3, CD4, CD8, CD19, CD56, CD45, and activation markers (Beijing Tongsheng Shidai Biotech Co. LTD). After staining and washing, cells were analyzed on a BECKMAN COULTER DxFLEX flow cytometer. Data were acquired and analyzed using CytExpert (Beckman, Inc.).

Lymphocyte subsets were defined as follows: CD3⁺CD4⁺ (helper T cells), CD3⁺CD8⁺ (cytotoxic T cells), CD3⁻CD19⁺ (B cells), CD3⁻CD56⁺ (natural killer cells), and activated T cells expressing CD25, CD69, or HLA-DR. The proportions of each subset were calculated as a percentage of total lymphocytes.

### RNA sequencing and data analyses

A total of 72 peripheral blood mononuclear cell (PBMC) samples were included in the transcriptomic analysis. This comprises paired pre- and post-treatment samples from the 20 patients in the GATES trial as described above (40 samples) and paired pre- and post-treatment samples from the 16 patients in the external control cohort (32 samples). The control cohort consisted of 16 patients with locally advanced HER2-negative G/EGJ adenocarcinoma, treated at our institution during 2024–2025 with the backbone regimen of serplulimab plus SOX, but without thymalfasin. The key selection criteria (clinical stage: cT3-4aN + M0; histology; HER2 status) were identical to those of the GATES trial to ensure population matching.

Paired-end RNA sequencing was performed on the Illumina platform. Raw reads were subjected to quality control and adapter trimming to generate high-quality clean reads. Alignment to the GRCh38 reference genome was conducted using STAR (v2.7.10b) in two-pass mode. Gene-level quantification was performed using featureCounts from the Subread package, and a gene symbol-based count matrix was constructed for downstream analysis. Differential expression analysis was performed using DESeq2 (v1.42.1) with a model incorporating treatment group, time point, and their interaction, while adjusting for sequencing depth and detected gene counts. Genes with |log_2_FC|> 1.5 and FDR < 0.1 were considered significant. Functional enrichment analysis of GO Biological Process and KEGG pathways was conducted using clusterProfiler (v4.10.1), with FDR < 0.05 as the significance threshold.

Weighted Gene Co-expression Network Analysis (WGCNA, v1.73) was applied to the variance-stabilized transformed expression matrix of the top 8000 most variable genes. A soft-thresholding power of 6 was selected to construct a signed co-expression network. Module-trait relationships were assessed by correlating module eigengenes with clinical variables, including treatment group, post-treatment time point, and pathological response. Immune cell fractions were estimated from bulk RNA-seq data using the quanTIseq algorithm via the immunedeconv (v2.1.0) R package, run in “non-tumor” mode.

### Statistical analysis

Statistical analysis was performed using SPSS, Graphpad Prism, and R for all intention to treat (ITT) populations. Continuous variables were summarized using medians and IQRs. Categorical variables were summarized with frequencies and percentages. Comparisons of patient characteristics between responders and nonresponders were performed using Fisher’s exact test. All tests were two-sided, with statistical significance defined at *p* < 0.05.

## Results

### Baseline characteristics of patients

Between June 2024 and December 2024, 32 patients with locally advanced G/EGJ adenocarcinoma were screened for eligibility at our center. Of these, 30 patients met the inclusion criteria and were enrolled in the GATES trial. The detailed inclusion and exclusion criteria are provided in Additional file 1. Baseline characteristics of the participants are summarized in Table 1.

**Table 1:** Baseline characteristics

Characteristics	Patients (*n* = 30)
**Age, years (median, IQR)**	65.5 (60.0–70.8)
≤ 65	15 (50.0)
> 65	15 (50.0)
**Sex**	
Female	3 (10.0)
Male	27 (90.0)
**Smoking**	
No	15 (50.0)
Yes	15 (50.0)
**Drinking**	
No	20 (66.7)
Yes	10 (33.3)
**Primary tumor location**	
Gastric	20 (66.7)
EGJ	10 (33.3)
***H.P* infection**	
No	9 (30.0)
Yes	21 (70.0)
**Tumor diameter, cm (median, IQR)**	5.0 (4.2–6.1)
≤ 5.0	15 (50.0)
> 5.0	15 (50.0)
**Histological differentiation**	
G2	13 (43.3)
G3	17 (56.7)
**Lauren type**	
Diffused	10 (33.3)
Intestinal	16 (53.3)
Mixed	4 (13.3)
**HER2 status**	
-	18 (60.0)
1 +	12 (40.0)
**DNA mismatch repair (MMR)**	
Proficient mismatch repair (pMMR)	28 (93.3)
Deficient mismatch repair (dMMR)	2 (6.7)
**PD-L1 status**	
CPS < 1	11 (36.7)
CPS 1–5	7 (23.3)
CPS ≥ 5	12 (40.0)
**Pretreated cT stage**	
T3	15 (50.0)
T4	15 (50.0)
**Pretreated cN stage**	
N2	17 (56.7)
N3	13 (43.3)
**Pretreated cTNM stage**	
IIIa	17 (56.7)
IIIb	13 (43.3)

Briefly, the median age of the cohort was 65.5 years (interquartile range [IQR], 60.0–70.8 years). Twenty patients (66.7%) had primary tumors in the stomach, while 10 patients (33.3%) had EGJ adenocarcinoma. The median tumor size at baseline was 5.0 cm (IQR, 4.2–6.1 cm). According to the Lauren classification, 16 patients (53.3%) were classified with the intestinal subtype, 10 (33.3%) with the diffuse subtype, and 4 (13.3%) with a mixed histological pattern. In terms of MMR status, 28 patients (93.3%) were proficient mismatch repair (pMMR), with only 2 (6.7%) identified as deficient mismatch repair (dMMR). For PD-L1 CPS, 11 patients (36.7%) had CPS < 1, 7 (23.3%) had CPS between 1 and 5, and 12 (40.0%) had CPS ≥ 5.

**Fig. 1:**
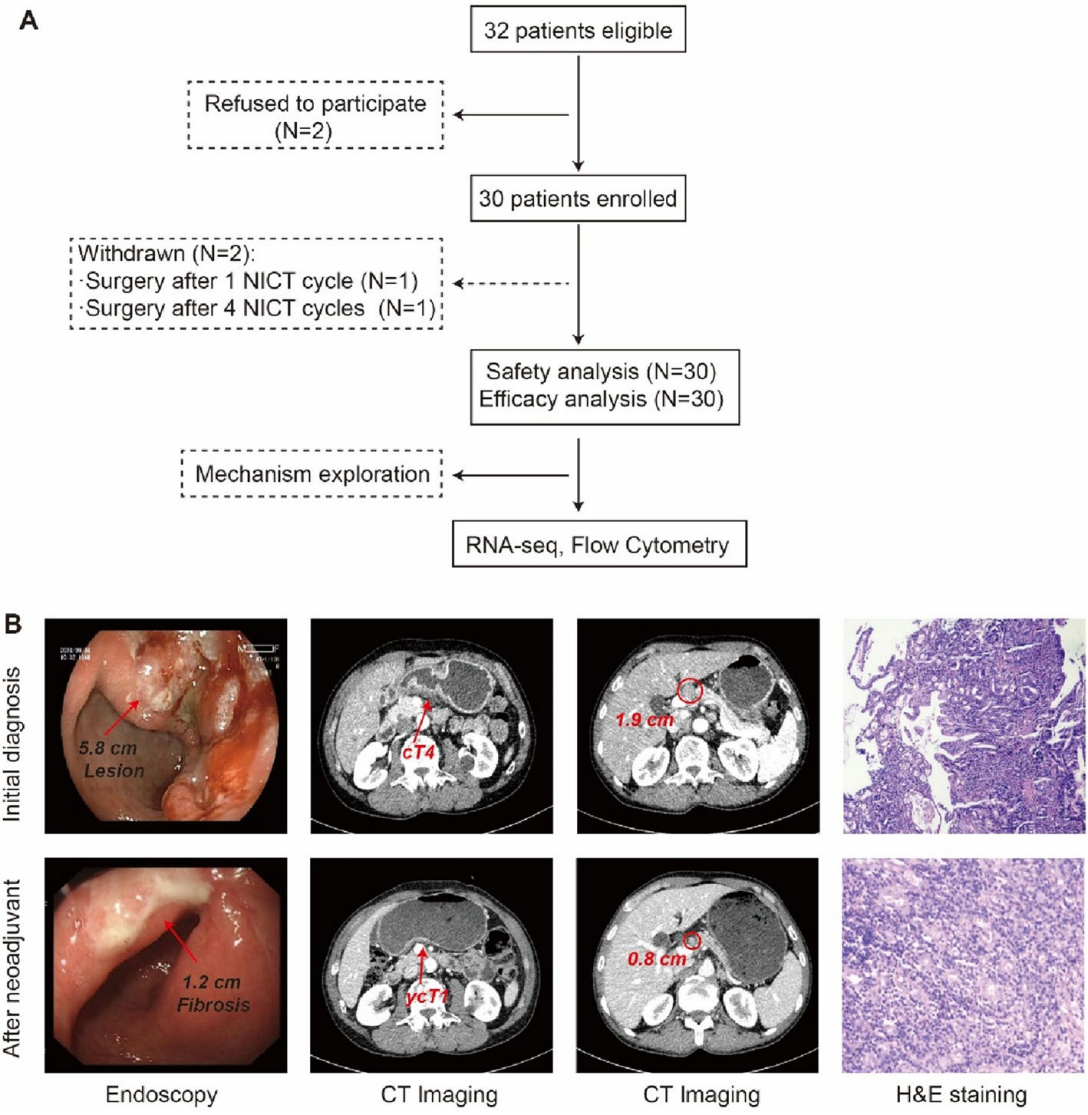
Flow diagram.. **A** Flow diagram of GATES trial. **B** Representative images at initial diagnosis and after NAT: endoscopic ulcerated lesion to fibrosis alteration; axial CT showing reduction of primary tumor (cT4 to ycT1) and nodal shrinkage; corresponding histopathology demonstrating treatment effect

Regarding the treatment course, one patient elected to undergo radical surgery after receiving only one cycle of neoadjuvant therapy, while another patient completed four cycles before surgery. The remaining 28 patients (93.3%) adhered to the designated three-cycle regimen prior to surgical intervention, as illustrated in Fig. 1.

### Safety profile

All enrolled patients (*n* = 30) were included in the safety analysis. Adverse events (AEs) and treatment-emergent adverse events (TEAEs) are summarized in Table 2. Nearly all patients (28/30, 93.3%) experienced at least one AE, while irAEs were observed in 7 patients (23.3%). Serious adverse events (SAEs), including treatment-emergent SAEs, occurred in 2 patients (6.7%). Grade 3 or higher AEs were reported in 8 patients (26.7%). Importantly, there were no treatment-related deaths or discontinuations due to toxicity. TEAEs leading to regimen modification were observed in 6 patients (20.0%): five required temporary treatment interruption, and one required a dose reduction of oxaliplatin. Notably, the patient who proceeded to surgery after only one cycle of therapy did so due to a combination of clinical judgment regarding optimal surgical timing and patient preference, and not as a direct consequence of a TEAE.

**Table 2:** Safety summary

Adverse events (AE),* n* (%)	Patients (*n* = 30)
Any AE	28 (93.3)
irAE	7 (23.3)
SAE	2 (6.7)
Treatment-emergent SAE	2 (6.7)
Grade ≥ 3 AE	8 (26.7)
Grade ≥ 3 TEAE	8 (26.7)
TEAE leading to discontinuation	0
TEAE leading to interruption	5 (16.7)
TEAE leading to reduction	1 (3.3)
TEAE leading to death	0

The spectrum of AEs is detailed in Table 3. The most frequently observed toxicities included hematological abnormalities: decreased platelet count (18/30, 60.0%), anemia (13/30, 43.3%), decreased white blood cell count (11/30, 36.7%), and decreased neutrophil count (11/30, 36.7%). Among these, grade ≥ 3 events were relatively uncommon, with severe thrombocytopenia (4/30, 13.3%) being the most notable and consistent with expected chemotherapy-induced myelosuppression. One of these patients required an additional hospitalization and was therefore classified as having experienced a SAE.

**Table 3:** All adverse events

Adverse events,* n* (%)	Patients (*n* = 30)		
	Any grade	Grades 1–2	Grade ≥ 3
Decreased platelet count	18 (60.0)	14 (46.7)	4 (13.3)
Anemia	13 (43.3)	12 (40.0)	1 (3.3)
Decreased white blood cell count	11 (36.7)	10 (33.3)	1 (3.3)
Decreased neutrophil count	11 (36.7)	9 (30.0)	2 (6.7)
Anorexia	11 (36.7)	11 (36.7)	0
Diarrhea	8 (26.7)	8 (26.7)	0
Dyspepsia	6 (20.0)	6 (20.0)	0
Vomiting	6 (20.0)	6 (20.0)	0
Nausea	5 (16.7)	5 (16.7)	0
Rash	4 (13.3)	4 (13.3)	0
Increased AST/ALT	4 (13.3)	4 (13.3)	0
Fatigue	3 (10.0)	3 (10.0)	0
Fever	2 (6.7)	2 (6.7)	0
Cough	2 (6.7)	2 (6.7)	0
Hypothyroidism	2 (6.7)	2 (6.7)	0
Hyperthyroidism	1 (3.3)	1 (3.3)	0
Hiccups	1 (3.3)	1 (3.3)	0
Constipation	1 (3.3)	1 (3.3)	0
Tinnitus	1 (3.3)	1 (3.3)	0
Skin hyperpigmentation	1 (3.3)	1 (3.3)	0
Gastrointestinal fistula	1 (3.3)	0	1 (3.3)

Non-hematological AEs were generally mild (grades 1–2) and predominantly included gastrointestinal symptoms such as anorexia (11/30, 36.7%), diarrhea (8/30, 26.7%). Severe non-hematological adverse events were rare, occurring only in 1 patient (3.3%) who developed a gastrointestinal fistula postoperatively and was successfully managed with reoperation. No life-threatening toxicities or irreversible organ damage were observed, reflecting a manageable safety profile for the regimen.

### Response to therapy

Radiographic assessment according to RECIST 1.1 criteria demonstrated encouraging outcomes (Table 4). Among the 30 patients evaluated, 3 (10.0%) achieved a complete response (CR) and 19 (63.3%) exhibited a partial response (PR). Stable disease (SD) was observed in 7 patients (23.3%), with no cases of progressive disease (PD) recorded. One patient (3.3%) did not undergo radiographic assessment due to early surgical intervention after only one treatment cycle. Collectively, the overall response rate (ORR) was 73.3% (95% CI, 56.5–90.1), and the disease control rate (DCR) reached 100%.

**Table 4:** Efficacy analyses

Outcomes,* n* (%)	Patients (*n* = 30)
**Radiographical assessment (RECIST 1.1)**	
Complete response (CR)	3 (10.0)
Partial response (PR)	19 (63.3)
Stable disease (SD)	7 (23.3)
Progressive disease (PD)	0 (0)
No assessment^a^	1 (3.3)
**Pathological T stage**	
ypT0	9 (30.0)
ypT1	1 (3.3)
ypT2	4 (13.3)
ypT3	10 (33.3)
ypT4	6 (20.0)
**Pathological N stage**	
ypN0	19 (63.3)
ypN1	5 (16.7)
ypN2	1 (3.3)
ypN3	5 (16.7)
**Pathological TNN stage**	
ypT0N0M0 (pCR)	9 (30.0)
ypTNM stage I	5 (16.7)
ypTNM stage II	9 (30.0)
ypTNM stage III	7 (23.3)
**Downstaging of T stage**	
No	10 (33.3)
Yes	20 (66.7)
**Downstaging of N stage**	
No	6 (20.0)
Yes	24 (80.0)
**Downstaging of TNM stage**	
No	7 (23.3)
Yes	23 (76.7)
**Major pathological response (MPR)**	
No	13 (43.3)
Yes	17 (56.7)

^a^No assessment included 1 patient who only received one neoadjuvant treatment and was unable to have his objective response evaluated by imaging

All patients underwent minimally invasive surgical approaches of R0 resection, including laparoscopic or robotic-assisted procedures (Additional file 2: Table S1). Surgical techniques comprised total gastrectomy (15/30, 50.0%), distal gastrectomy (13/30, 43.3%), and proximal gastrectomy (2/30, 6.7%). The total dissected lymph nodes were 52 (IQR, 38–61.8). The median intraoperative blood loss was 50 mL (IQR, 30–65 mL), and the median operative time was 206 min (IQR, 177–226 min). Following surgery, the median postoperative hospital stay was 7 days (IQR, 5–8 days). Perioperative complications, graded according to the Dindo-Demartines-Clavien classification, occurred in 3 patients (10.0%): one case each of grade II pulmonary infection, grade II pleural effusion, and grade IIIb gastrointestinal fistula (requiring reoperation). No perioperative mortality was observed.

Pathological assessment revealed a pCR (ypT0N0M0) in 9 patients (30.0%) and MPR was achieved in 17 patients (56.7%). Notably, 19 patients (63.3%, 95% CI, 45.0–81.6) achieved ypN0 status, indicating the complete eradication of detectable lymph node metastasis by neoadjuvant regimens, underscoring the effectiveness of this approach in reducing both primary tumor burden and lymphatic involvement (Table 4).

**Fig. 2:**
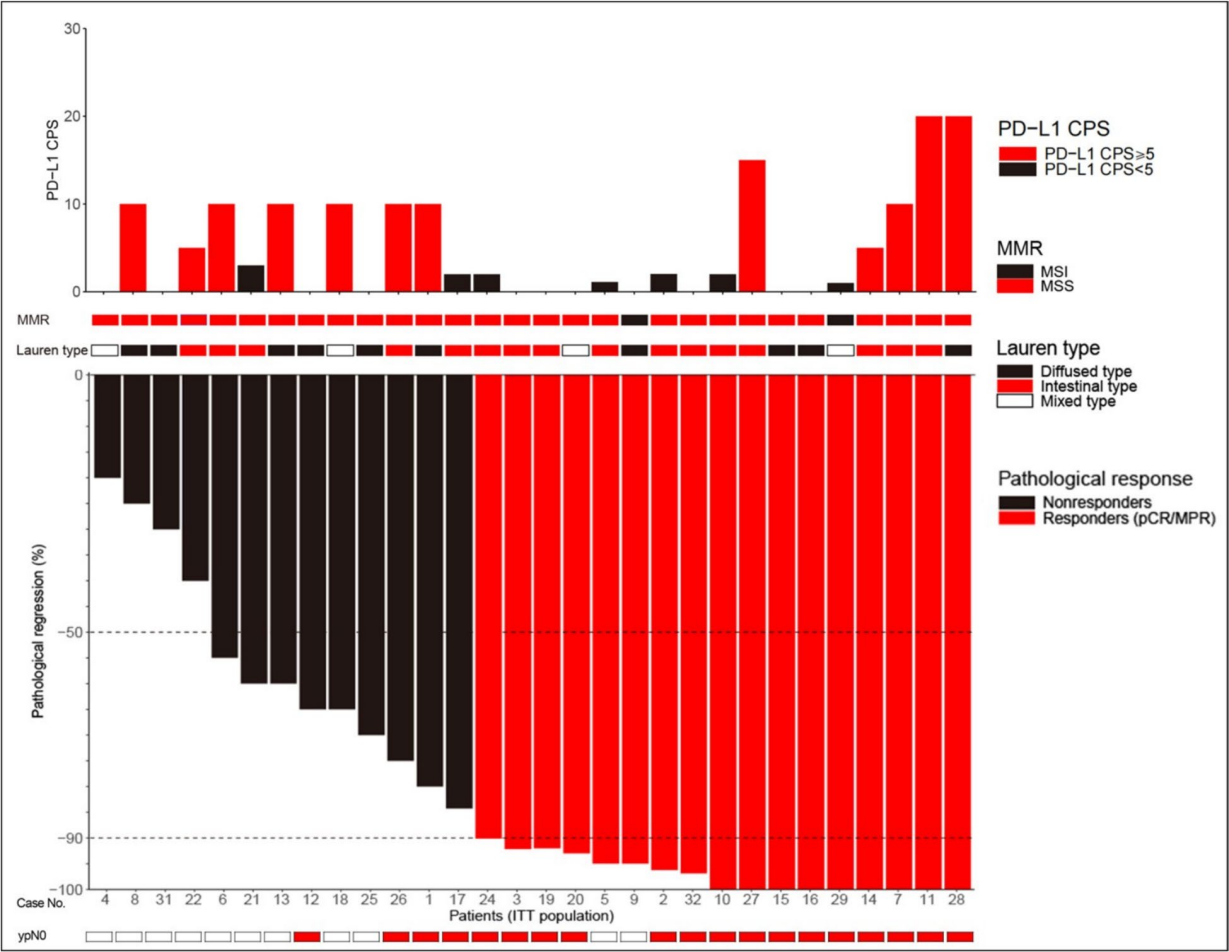
Pathological response in ITT population. Waterfall plot shows the association between tumor pathological regression and PD-L1 CPS, MMR status, and Lauren type. The numbers at the bottom represent case number. Abbreviations: ITT, intention to treat; PD-L1 CPS, programmed death-ligand 1 combined positive score; dMMR, deficient mismatch repair; pMMR, proficient mismatch repair; pCR, pathological complete response; MPR, major pathological response

We further explored the associations between pathological response and PD-L1 expression levels. Interestingly, pMMR patients with CPS < 1 still achieved a pCR rate of 18.2% (2/11), indicating treatment efficacy even in traditionally less responsive subgroups. Among patients with CPS ≥ 1, the pCR rate was 36.8% (7/19). Although numerically higher, the difference did not reach statistical significance (*P* = 0.419). The detailed information of each patient is exhibited in Fig. 2.

Analysis of MPR rates similarly showed no significant association with PD-L1 status (*P* = 0.379). The rates of MPR were 63.6% (7/11) for CPS < 1, 71.4% (5/7) for CPS 1–5, and 41.7% (5/12) for CPS ≥ 5, reflecting no clear CPS-dependent pattern. Given the substantial ypN0 downstaging observed, we also analyzed its correlation with PD-L1 CPS scores. Consistent with the MPR findings, no CPS-dependent trend emerged: the ypN0 downstaging rates were 63.6% (7/11) for CPS < 1, 71.4% (5/7) for CPS 1–5, and 58.3% (7/12) for CPS ≥ 5.

Additionally, subgroup analyses revealed no significant differences in pCR or MPR based on other clinical and pathological variables, including tumor location, *H. pylori* infection status, tumor size, histological differentiation, Lauren classification, and pre-treatment clinical stage (Table 5). These findings suggest that thymalfasin combined with neoadjuvant immunochemotherapy may enhance therapeutic responses independently of traditional biomarkers, potentially broadening its clinical applicability.

**Table 5:** Comparison of patients’ characteristics between responders (pCR or MPR) and nonresponders

Characteristics	Total (*n* = 30)	pCR or MPR (*n* = 17)	Others (*n* = 13)	*P* value
**Age, years**				
≤ 65	15	9	6	1.000
> 65	15	8	7	
**Sex**				
Female	3	1	2	0.565
Male	27	16	11	
**Smoking**				
No	15	10	5	0.462
Yes	15	7	8	
**Drinking**				
No	20	13	7	0.255
Yes	10	4	6	
**Tumor location**				
Gastric cancer	20	11	9	1.000
EGJ cancer	10	6	4	
***H.P* infection**				
No	9	5	4	1.000
Yes	21	12	9	
**Tumor diameter, cm**				
≤ 5.0	15	10	5	0.462
> 5.0	15	7	8	
**Differentiation grade**				
G2	13	8	5	0.721
G3	17	9	8	
**Lauren classification**				
Diffused	10	4	6	0.340
Intestinal	16	11	5	
Mixed	4	2	2	
**HER2 status**				
-	18	10	8	1.000
1 +	12	7	5	
**DNA mismatch repair**				
pMMR	28	15	13	0.492
dMMR	2	2	0	
**PD-L1 status**				
CPS < 1	11	7	4	0.379
CPS 1–5	7	5	2	
CPS ≥ 5	12	5	7	
**Pretreated cT stage**				
T3	15	9	6	1.000
T4	15	8	7	
**Pretreated cN stage**				
N2	17	9	8	0.721
N3	13	8	5	
**Pretreated cTNM stage**				
IIIa	17	9	8	0.721
IIIb	13	8	5	

### Peripheral blood lymphocyte subset dynamics

**Fig. 3:**
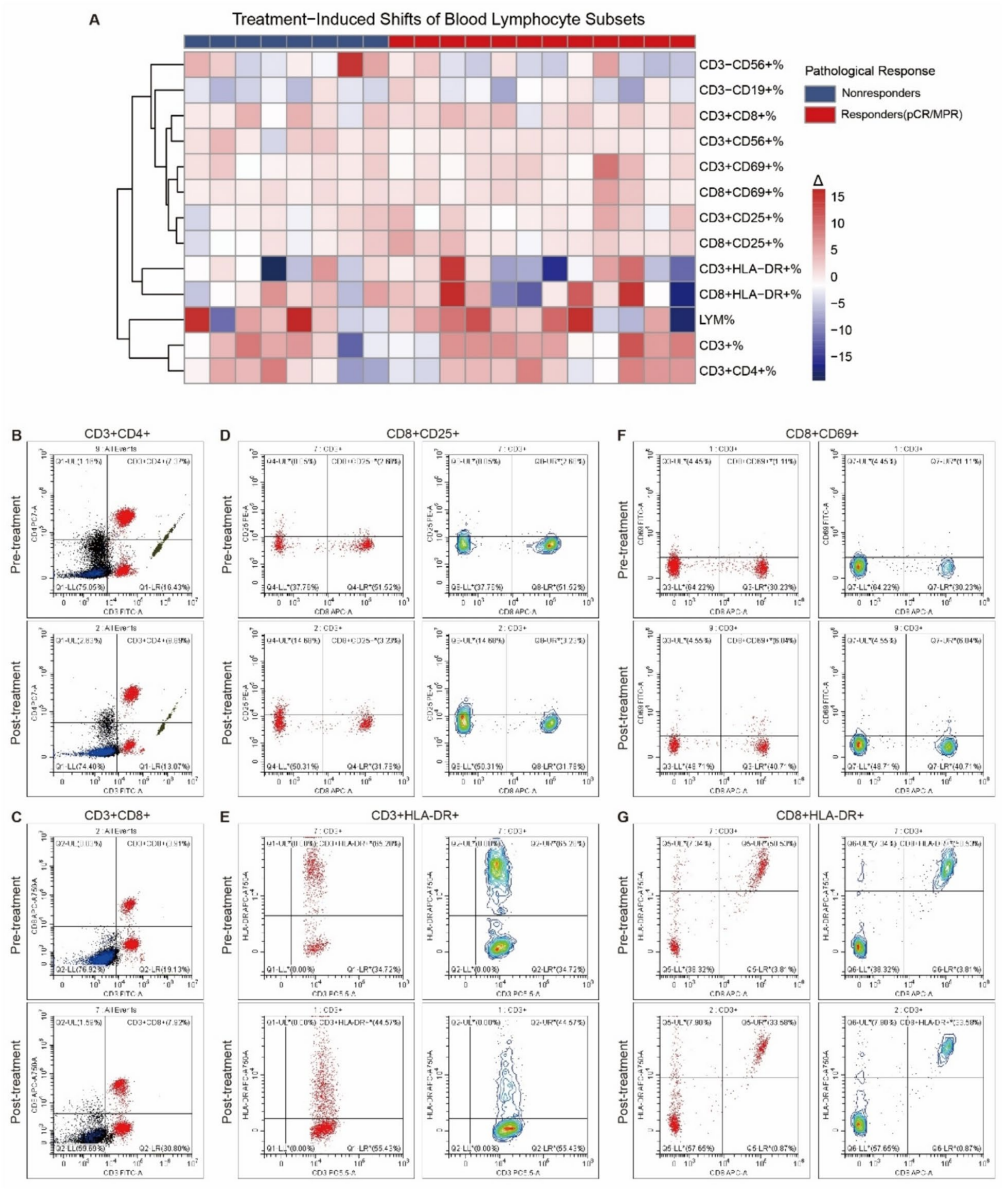
Changes in peripheral blood lymphocyte subsets before and after neoadjuvanttherapy. Peripheral blood samples were collected from 20 patients at baseline and after completion of neoadjuvant treatment. **A** Flow cytometric analysis was performed to evaluate the proportions of key lymphocyte subsets, including CD3⁻CD56⁺ natural killer (NK) cells, CD3⁻CD19⁺ B cells, CD3⁺CD4⁺ and CD3⁺CD8⁺ T cells, and activated lymphocyte populations marked by CD69, CD25, and HLA-DR expression. The heatmap illustrates the dynamic changes (Δ) in the percentages of these subsets following treatment. Patients were stratified into responders (pCR/MPR) and nonresponders based on pathological response. **B**–**G** Paired representative flow cytometry plots depict treatment-induced shifts of major lymphocyte subsets: **B** CD3⁺CD4⁺, **C** CD3⁺CD8⁺, **D** CD8⁺CD25⁺, **E** CD3⁺HLA-DR⁺, **F** CD8⁺CD69⁺, and **G** CD8⁺ HLA-DR⁺ cells. Upper and lower panels show pre- and post-treatment status, respectively

To explore the immunological impact of the combination therapy, we analyzed peripheral blood lymphocyte subsets in 20 patients at two timepoints: pre-treatment and post-neoadjuvant therapy. Flow cytometry revealed distinct alterations in lymphocyte composition across several subsets (Fig. 3). Notably, there was an expansion of CD8⁺ T cells and increased expression of the early activation marker CD69 on CD8⁺ cells, consistent with robust initial T-cell activation and clonal expansion. In contrast, the proportion of T cells expressing the late activation marker HLA-DR decreased within both the CD3⁺ and CD8⁺ compartments. This seemingly paradoxical pattern—heightened early activation markers alongside reduced HLA-DR expression—suggests a transition from a state of broad systemic activation to a more focused effector or memory-oriented response. Activated T cells may be trafficking to tumor or nodal sites (antigen-directed homing) or differentiating into longer-lived memory subsets, processes that are not directly captured in peripheral blood.

CD4⁺ T-cell proportions and CD25⁺ mid-term activation markers remained relatively stable, implying that the immune response was enhanced but not globally dysregulated. In exploratory analyses, patients achieving pCR or MPR tended to show more pronounced increases in activated CD8⁺ subsets than non-responders, suggesting a correlation between systemic immune activation and pathological response. Additionally, a decrease in B cell (CD3⁻CD19⁺) proportion was observed in several sensitive cases, which may be associated with reduced humoral suppression.

### Peripheral blood transcriptomic profiling

**Fig. 4:**
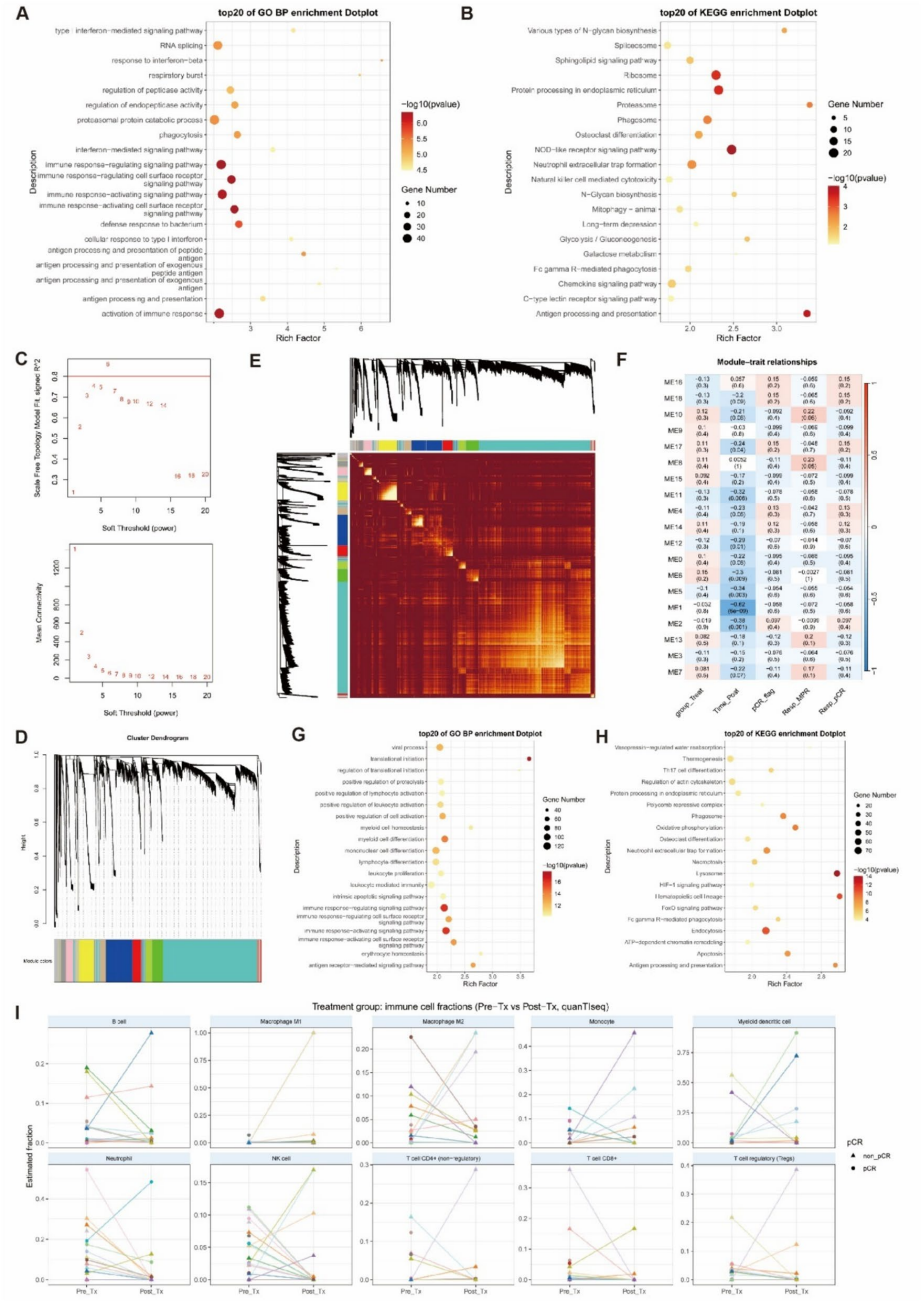
Weighted Gene Co-expression Network Analysis (WGCNA) and deconvolution of PBMC transcriptomes. **A**, **B** GO Biological process (**A**) and KEGG pathway (**B**) enrichment results for upregulated genes from the treatment-by-time interaction analysis. **C** Scale-free topology model fit for WGCNA soft-thresholding power selection. A power of *β* = 6 was chosen for network construction. **D** Gene dendrogram and module assignment from WGCNA. Colored bars indicate co-expression modules. **E** Heatmap of the topological overlap matrix (TOM) for all genes included in WGCNA, with module assignments shown. **F** Module–trait relationships showing Pearson correlations between module eigengenes and clinical variables. Values indicate correlation coefficients (with adjusted *p*-values in parentheses). **G**, **H** GO (**H**) and KEGG (**I**) enrichment analyses for genes in the ME1 module. **I** Paired changes in immune cell fractions estimated by quanTIseq, stratified by pathological response (pCR vs. non-pCR)

Bulk RNA-seq of PBMCs from thymalfasin-treated and control immunochemotherapy cohorts provided complementary insight into systemic immune remodeling. To ensure the validity of the comparative transcriptomic analysis, we first confirmed that the patients in the PBMC/RNA-seq subset (*n* = 20) were representative of the entire GATES trial population (*n* = 30), with no significant differences in key baseline characteristics or primary efficacy endpoints (all *P* > 0.05; Additional file 3: Table S2). The external control cohort (*n* = 16), comprised of patients treated with serplulimab plus SOX (without thymalfasin) was well-matched to the GATES RNA-seq subset in demographics, clinical stage, PD-L1 CPS, and MMR status (all *P* > 0.05). All samples were processed and sequenced in a single batch. While the cohorts were balanced at baseline, the GATES regimen, as anticipated, induced a significantly higher rate of pathological nodal clearance (ypN0: 80.0% vs. 37.5%, *P* = 0.009), reflecting its therapeutic efficacy. Therefore, the between-group differential expression analysis is presented as exploratory, and mechanistic interpretation is primarily anchored to the paired pre-/post-treatment analyses within the GATES cohort to identify thymalfasin-specific immunological changes. Differential expression analysis focusing on the treatment-by-time interaction identified genes upregulated over the treatment course in the thymalfasin group relative to controls. As shown in Fig. 4A, B, these genes were enriched for GO and KEGG pathways related to antigen processing and presentation, type I interferon signaling, innate immune sensing, phagocytosis and neutrophil extracellular trap formation, proteasome-mediated protein degradation, and immune-metabolic remodeling (including glycolysis). Together, these signatures suggest that thymalfasin reinforces key steps of the cancer-immunity cycle, from antigen capture and presentation to effector cell priming and metabolic support.

WGCNA identified a co-expression module (ME1) heavily enriched for these immune activation pathways (Fig. 4C–E). Interestingly, ME1 eigengene values declined over time in the overall cohort, consistent with a general contraction of systemic immune activation as tumor burden decreases and immune cells redistribute. However, this decline was attenuated in the thymalfasin group, indicating relative preservation of antigen-presentation and interferon signaling programs compared with controls (Fig. 4F–H). QuanTIseq-based deconvolution revealed treatment-associated changes in immune cell composition, including trends in T-cell and myeloid populations that differed between responders and non-responders, although patterns were heterogeneous in this modest-sized dataset (Fig. 4I).

### Survival and follow-up

**Fig. 5:**
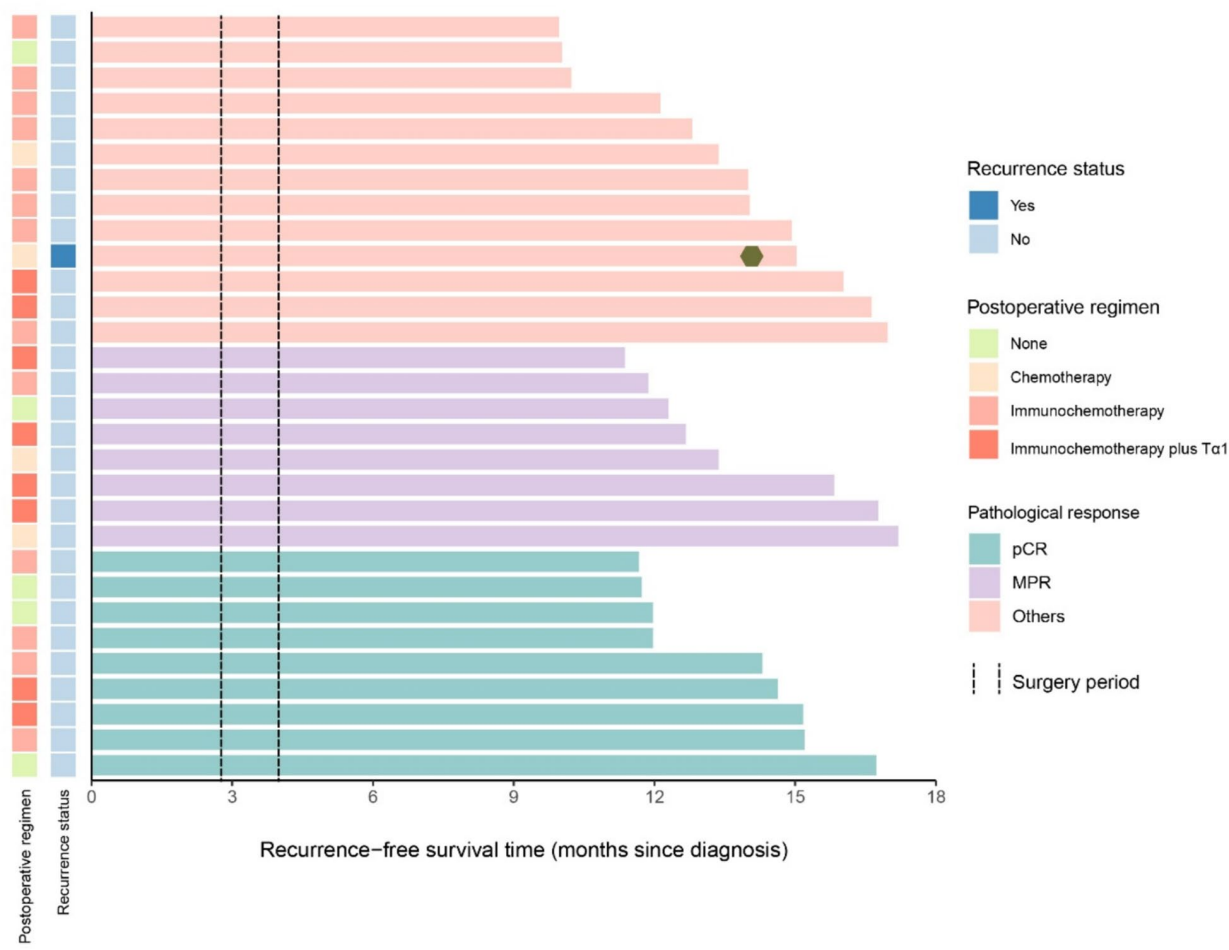
Survival analyses of all enrolled cases. Swimmer plot illustrating relapse-free survival and overall survival for all enrolled patients, with individual bars annotated according to postoperative treatment regimen and pathological response status

After surgery, 83.3% (25/30) patients received an additional three cycles of adjuvant therapy: 8 continued on immunochemotherapy plus thymalfasin, 13 received immunochemotherapy alone, and 4 were treated with chemotherapy only. At the data cut-off date, the median follow-up duration was 14.0 months (range 10.0–17.2 months). During this period, only one patient experienced disease recurrence—a retroperitoneal lymph node relapse at 14.4 months after diagnosis. No deaths had occurred at the time of analysis (Fig. 5). Although follow-up remains relatively short and the number of events is limited, these early data suggest promising disease control following this neoadjuvant regimen.

## Discussion

Clinical trials targeting advanced G/EGJ adenocarcinoma have consistently demonstrated that only patients with high PD-L1 expression derive substantial benefit from immune checkpoint inhibitors [26–28]. Similar outcomes have been observed in the neoadjuvant setting for locally advanced disease, where pathological responses in primary lesions and metastatic lymph nodes remain unsatisfactory. In this context, the GATES trial represents the first study to evaluate the synergistic effect of thymalfasin, an immune modulator, in combination with neoadjuvant immunochemotherapy for locally advanced G/EGJ adenocarcinoma. Our results indicate promising efficacy and a favorable safety profile, with a pCR achieved in 30.0% of patients and a MPR in 56.7%. Most notably, lymph node metastasis was effectively targeted, with 63.3% of patients achieving ypN0 status and 80.0% experiencing overall nodal downstaging.

Previous neoadjuvant immunochemotherapy trials in gastric cancer, such as KEYNOTE-585 and NEOSUMMIT-01, have reported varying lymph node clearance rates. In KEYNOTE-585, the N0 downstaging rate was 29.9% in the immunochemotherapy group, markedly higher than the 17.5% observed in the chemotherapy-only group [13]. Conversely, the NEOSUMMIT-01 trial reported no significant difference in N0 downstaging between the immunochemotherapy (40.7%) and chemotherapy (38.9%) groups [29]. Modest outcomes were also observed in the DRAGON IV/CAP 05 study, where N0 downstaging rates reached 46% in the camrelizumab plus rivoceranib plus SOX group, compared to 37% with SOX alone [30]. Furthermore, in a separate study comparing camrelizumab and apatinib combined with nab-paclitaxel plus S-1 (CA-SAP group) versus chemotherapy alone (SAP group), the N0 downstaging rates were 25.5% and 17.0%, respectively [31]. The pronounced ypN0 rate in our trial (63.3%) suggests that incorporating thymalfasin into neoadjuvant immunochemotherapy may be particularly effective at clearing nodal micrometastases. This clinical observation is consistent with our peripheral immune profiling, in which flow cytometry revealed an expansion of CD8⁺ T cells and increased CD69 expression—hallmarks of early activation and clonal expansion—together with a decline in HLA-DR-positive T cells within the CD3⁺ and CD8⁺ compartments. These changes support a model in which thymalfasin-augmented immunochemotherapy triggers an initial wave of broad CD8⁺ T-cell activation that subsequently narrows into a more focused pool of effector and memory cells, likely after trafficking to tumor and nodal sites. Such coordinated T cell-mediated immunity may help explain the high rate of nodal sterilization and could mitigate resistance mechanisms that limit the efficacy of conventional neoadjuvant immunochemotherapy.

In the KEYNOTE-585 study, response rates were significantly associated with higher PD-L1 expression. Specifically, patients with CPS ≥ 1 who received immunochemotherapy achieved a 12.1% higher pCR rate compared to those treated with chemotherapy alone, whereas no significant difference was observed in patients with CPS < 1 [13]. Similarly, the NEOSUMMIT-01 trial reported pCR rates of 45.5% in patients with CPS ≥ 5, compared to only 15.6% in those with CPS < 5 undergoing immunochemotherapy [29]. Consistent with this trend, a phase II neoadjuvant study using sintilimab combined with CapeOx (oxaliplatin and capecitabine) demonstrated a pCR rate of 28.6% in patients with CPS ≥ 1, contrasting with just 9.1% in those with CPS < 1 [32]. Additional neoadjuvant trials, such as DRAGON IV/CAP 05, SHARED, and MATTERHORN, have similarly highlighted PD-L1 expression as a key determinant of therapeutic response [30, 31, 33–35].

In our cohort, the pCR rate reached 36.8% among patients with PD-L1 CPS ≥ 1. Notably, even in the CPS < 1 subgroup, the pCR rate was 18.2%, indicating that thymalfasin may augment antitumor immune activity in patients who are traditionally viewed as less responsive to immunotherapy. This broader pattern of efficacy is consistent with the notion that the addition of thymalfasin to immunochemotherapy may help mitigate the limitations associated with low PD-L1 expression and could extend the therapeutic benefit of neoadjuvant immunochemotherapy to CPS < 1 patients, who are often underrepresented or excluded in conventional immunotherapy protocols [36, 37].

Transcriptomic profiling further revealed immune signatures associated with the thymalfasin-containing regimen that reinforce the concept of thymalfasin-enhanced systemic immunity. In the thymalfasin group, upregulated genes were enriched for pathways related to antigen processing and presentation, type I interferon signaling, etc. WGCNA identified a co-expression module encompassing these functions that showed an overall decline with treatment—consistent with decreasing tumor burden and immune contraction—but remained relatively preserved in thymalfasin-treated patients. This pattern suggests that thymalfasin helps maintain key components of the antitumor immune cascade during the neoadjuvant window, supporting durable immune surveillance without driving persistent, harmful hyperinflammation.

Safety remains a critical concern in immunochemotherapy regimens [38]. Previous studies have suggested a positive correlation between the occurrence and severity of irAEs and the clinical efficacy of immune checkpoint blockade, particularly in the neoadjuvant setting. Patients who develop irAEs often exhibit more robust immune activation, which may translate into improved pathological responses. However, this enhanced immune activity also raises concerns about toxicity, especially when combined with cytotoxic chemotherapy. Based on this, we hypothesized that the integration of thymalfasin into neoadjuvant immunochemotherapy could provide a dual benefit on maintaining or even enhancing antitumor efficacy while simultaneously minimizing the risk of irAEs. Our results support this hypothesis with an overall low incidence of irAEs at 23.3%, compared to almost 70% observed in camrelizumab plus mFOLFOX6 therapy [34] or even higher in the Neo-PLANET trial [39]. Of note, there is no grade 3 or 4 irAE observed in our study, compared to the 7.2% in MATTERHORN or even higher in other trials [35]. This favorable safety profile may reflect the immunomodulatory nature of thymalfasin, which appears to fine-tune rather than indiscriminately amplify immune activation.

Our peripheral data are consistent with this concept. As discussed above, flow cytometry data suggests robust but controlled T-cell activation, rather than systemic hyperactivation, excessive cytokine release, and B cell-driven autoantibody production that typically underlie severe irAEs [40]. In this context, the inclusion of thymalfasin in the regimen may be associated with a reshaping of the peripheral immune landscape in a way that preserves antitumor activity while limiting off-target toxicity—a profile that is particularly attractive in the perioperative setting, where potent yet tightly regulated immune engagement is required. However, several limitations should be considered when interpreting our findings.

First, the single-arm, non-randomized design precludes direct causal attribution of the observed efficacy to the addition of thymalfasin and limits comparative effectiveness conclusions. Second, the sample size, though appropriate for this phase II trial, is modest and may affect the generalizability of the results. Third, heterogeneity existed in the postoperative adjuvant treatment strategies, which could influence long-term survival outcomes. These factors underscore that our results, while encouraging, require validation in larger, randomized controlled trials. Fourth, the chemotherapeutic backbone of SOX is predominantly utilized in East Asia. While it is a standard and highly effective regimen in this context, its limited adoption in other regions may affect the generalizability of our findings to populations where different chemotherapy backbones are the standard of care.

## Conclusions

Neoadjuvant serplulimab, SOX chemotherapy, and thymalfasin achieved high pCR and MPR rates, deep nodal clearance, and encouraging early disease control with acceptable toxicity in patients with HER2-negative stage III gastric and gastroesophageal junction adenocarcinoma. Peripheral immune profiling and PBMC transcriptomics revealed immune patterns that are compatible with a model wherein thymalfasin, as part of the combination, could contribute to enhanced and stabilized systemic antitumor immunity while being associated with a manageable toxicity profile. These results justify further randomized evaluation of thymalfasin-augmented neoadjuvant immunochemotherapy and suggest that this approach may help extend the benefits of PD-1 blockade beyond PD-L1-selected subgroups.

## Supplementary Information

Additional file 1. Study protocol.

Additional file 2: Table S1. Perioperative and surgical outcomes.

Additional file 3: Table S2. Baseline characteristics and comparability of the study cohorts.

Additional file 4. CONSORT checklist.

## Data Availability

The study protocol is provided as Additional file 1. Deidentified individual participant data (including data underlying the primary outcome results) and a data dictionary defining each field in the dataset will be made available upon reasonable request to the corresponding author. The raw RNA sequencing data reported in this paper have been deposited in the Genome Sequence Archive [41] in National Genomics Data Center [42], China National Center for Bioinformation/Beijing Institute of Genomics, Chinese Academy of Sciences (GSA-Human: HRA016845) that are publicly accessible at https://ngdc.cncb.ac.cn/gsa-human. Researchers must submit a methodologically sound proposal, and data will be shared for academic, non-commercial purposes only. There are no additional restrictions on data use beyond those stated.

## Abbreviations

AE: Adverse Event
AJCC: American Joint Committee on Cancer
CPS: Combined positive score
CR: Complete response
CTCAE: Common Terminology Criteria for Adverse Events
CTL: Cytotoxic T lymphocyte
DCR: Disease control rate
dMMR: Deficient mismatch repair
ECOG: Eastern Cooperative Oncology Group
EGJ: Gastroesophageal junction
G/EGJ: Gastric and gastroesophageal junction
HER2: Human epidermal growth factor receptor 2
HLA-DR: Human leukocyte antigen-DR isotype
irAE: Immune-related adverse event
IQR: Interquartile range
ITT: Intention to treat
MRI: Magnetic resonance imaging
MPR: Major pathological response
NCCN: National Comprehensive Cancer Network
NK: Natural killer
ORR: Objective response rate
PBMC: Peripheral blood mononuclear cell
pCR: Pathological complete response
PD: Progressive disease
PD-1: Programmed death receptor-1
PD-L1: Programmed death-ligand 1
pMMR: Proficient mismatch repair
PR: Partial response
RFS: Relapse-free survival
RNA-seq: RNA sequencing
SAE: Serious adverse event
SD: Stable disease
SOX: S-1 and oxaliplatin
TEAE: Treatment–emergent adverse event
TLR: Toll-like receptor
TRG: Tumor regression grade
WGCNA: Weighted Gene Co-expression Network Analysis

## References

[1] Siegel RL, Kratzer TB, Giaquinto AN, Sung H, Jemal A. Cancer statistics, 2025. CA Cancer J Clin. 2025;75(1):10–45. 39817679 10.3322/caac.21871 PMC11745215

[2] Han B, Zheng R, Zeng H, Wang S, Sun K, Chen R, et al. Cancer incidence and mortality in China, 2022. J Natl Cancer Cent. 2024;4(1):47–53. 39036382 10.1016/j.jncc.2024.01.006 PMC11256708

[3] Lordick F, Carneiro F, Cascinu S, Fleitas T, Haustermans K, Piessen G, et al. Gastric cancer: ESMO clinical practice guideline for diagnosis, treatment and follow-up. Ann Oncol. 2022;33(10):1005–20. 35914639 10.1016/j.annonc.2022.07.004

[4] Japanese Gastric Cancer Association. Japanese gastric cancer treatment guidelines 2021 (6th edition). Gastric Cancer. 2023;26(1):1–25. 10.1007/s10120-022-01331-8 PMC9813208 36342574

[5] Bang YJ, Kim YW, Yang HK, Chung HC, Park YK, Lee KH, et al. Adjuvant capecitabine and oxaliplatin for gastric cancer after D2 gastrectomy (CLASSIC): a phase 3 open-label, randomised controlled trial. Lancet. 2012;379(9813):315–21. 22226517 10.1016/S0140-6736(11)61873-4

[6] Japanese Gastric Cancer Association. Japanese gastric cancer treatment guidelines 2025 (7th edition). Gastric Cancer (2026). 10.1007/s10120-025-01698-4. 10.1007/s10120-025-01698-4 PMC12956939 41569370

[7] National Comprehensive Cancer Network. NCCN Guidelines for Gastric Cancer V.2.2025. https://www.nccn.org/guidelines/guidelines-detail?category=1&id=1434 (accessed May 25, 2025).

[8] Kang YK, Yook JH, Park YK, Lee JS, Kim YW, Kim JY, et al. PRODIGY: a phase iii study of neoadjuvant docetaxel, oxaliplatin, and S-1 plus surgery and adjuvant S-1 versus surgery and adjuvant S-1 for resectable advanced gastric cancer. J Clin Oncol. 2021;39(26):2903–13. 34133211 10.1200/JCO.20.02914 PMC8425847

[9] Zhang X, Liang H, Li Z, Xue Y, Wang Y, Zhou Z, et al. Perioperative or postoperative adjuvant oxaliplatin with S-1 versus adjuvant oxaliplatin with capecitabine in patients with locally advanced gastric or gastro-oesophageal junction adenocarcinoma undergoing D2 gastrectomy (RESOLVE): an open-label, superiority and non-inferiority, phase 3 randomised controlled trial. Lancet Oncol. 2021;22(8):1081–92. 34252374 10.1016/S1470-2045(21)00297-7

[10] Marin-Acevedo JA, Kimbrough EO, Lou Y. Next generation of immune checkpoint inhibitors and beyond. J Hematol Oncol. 2021;14(1):45. 33741032 10.1186/s13045-021-01056-8 PMC7977302

[11] Verschoor YL, van de Haar J, van den Berg JG, van Sandick JW, Kodach LL, van Dieren JM, et al. Neoadjuvant atezolizumab plus chemotherapy in gastric and gastroesophageal junction adenocarcinoma: the phase 2 PANDA trial. Nat Med. 2024;30(2):519–30. 38191613 10.1038/s41591-023-02758-x PMC10878980

[12] Holder AM, Dedeilia A, Sierra-Davidson K, Cohen S, Liu D, Parikh A, et al. Defining clinically useful biomarkers of immune checkpoint inhibitors in solid tumours. Nat Rev Cancer. 2024;24(7):498–512. 38867074 10.1038/s41568-024-00705-7

[13] Shitara K, Rha SY, Wyrwicz LS, Oshima T, Karaseva N, Osipov M, et al. Neoadjuvant and adjuvant pembrolizumab plus chemotherapy in locally advanced gastric or gastro-oesophageal cancer (KEYNOTE-585): an interim analysis of the multicentre, double-blind, randomised phase 3 study. Lancet Oncol. 2024;25(2):212–24. 38134948 10.1016/S1470-2045(23)00541-7

[14] Shitara K, Ajani JA, Moehler M, Garrido M, Gallardo C, Shen L, et al. Nivolumab plus chemotherapy or ipilimumab in gastro-oesophageal cancer. Nature. 2022;603(7903):942–8. 35322232 10.1038/s41586-022-04508-4 PMC8967713

[15] Rha SY, Oh DY, Yañez P, Bai Y, Ryu MH, Lee J, et al. Pembrolizumab plus chemotherapy versus placebo plus chemotherapy for HER2-negative advanced gastric cancer (KEYNOTE-859): a multicentre, randomised, double-blind, phase 3 trial. Lancet Oncol. 2023;24(11):1181–95. 37875143 10.1016/S1470-2045(23)00515-6

[16] Conlon KC, Lugli E, Welles HC, Rosenberg SA, Fojo AT, Morris JC, et al. Redistribution, hyperproliferation, activation of natural killer cells and CD8 T cells, and cytokine production during first-in-human clinical trial of recombinant human interleukin-15 in patients with cancer. J Clin Oncol. 2015;33(1):74–82. 25403209 10.1200/JCO.2014.57.3329 PMC4268254

[17] Xue L, Thatte AS, Mai D, Haley RM, Gong N, Han X, et al. Responsive biomaterials: optimizing control of cancer immunotherapy. Nat Rev Mater. 2024;9(2):100–18.

[18] Romani L, Moretti S, Fallarino F, Bozza S, Ruggeri L, Casagrande A, et al. Jack of all trades: thymosin α1 and its pleiotropy. Ann N Y Acad Sci. 2012;1269:1–6. 23045964 10.1111/j.1749-6632.2012.06716.x

[19] Wei YT, Wang XR, Yan C, Huang F, Zhang Y, Liu X, et al. Thymosin α-1 reverses M2 polarization of tumor-associated macrophages during efferocytosis. Cancer Res. 2022;82(10):1991–2002. 35364609 10.1158/0008-5472.CAN-21-4260

[20] Liu F, Qiu B, Xi Y, Luo Y, Luo Q, Wu Y, et al. Efficacy of thymosin α1 in management of radiation pneumonitis in patients with locally advanced non-small cell lung cancer treated with concurrent chemoradiotherapy: a phase 2 clinical trial (GASTO-1043). Int J Radiat Oncol Biol Phys. 2022;114(3):433–43. 35870709 10.1016/j.ijrobp.2022.07.009

[21] Danielli R, Cisternino F, Giannarelli D, Calabrò L, Camerini R, Savelli V, et al. Long-term follow up of metastatic melanoma patients treated with thymosin alpha-1: investigating immune checkpoints synergy. Expert Opin Biol Ther. 2018;18(sup1):77–83. 30063847 10.1080/14712598.2018.1494717

[22] Garaci E, Pica F, Matteucci C, Gaziano R, D’Agostini C, Miele MT, et al. Historical review on thymosin α1 in oncology: preclinical and clinical experiences. Expert Opin Biol Ther. 2015;15(Suppl 1):S31–9. 26096345 10.1517/14712598.2015.1017466

[23] Liu K, Kong L, Cui H, Zhang L, Xin Q, Zhuang Y, et al. Thymosin α1 reverses oncolytic adenovirus-induced M2 polarization of macrophages to improve antitumor immunity and therapeutic efficacy. Cell Reports Medicine. 2024;5(10):101751. 39357524 10.1016/j.xcrm.2024.101751 PMC11513825

[24] Ni C, Wu P, Wu X, Zhang T, Zhang T, Wang Z, et al. Thymosin alpha1 enhanced cytotoxicity of iNKT cells against colon cancer via upregulating CD1d expression. Cancer Lett. 2015;356(2 Pt B):579–88. 25304368 10.1016/j.canlet.2014.10.002

[25] Shrivastava P, Singh SM, Singh N. Effect of thymosin alpha 1 on the antitumor activity of tumor-associated macrophage-derived dendritic cells. J Biomed Sci. 2004;11(5):623–30. 15316138 10.1007/BF02256128

[26] Janjigian YY, Shitara K, Moehler M, Garrido M, Salman P, Shen L, et al. First-line nivolumab plus chemotherapy versus chemotherapy alone for advanced gastric, gastro-oesophageal junction, and oesophageal adenocarcinoma (CheckMate 649): a randomised, open-label, phase 3 trial. Lancet. 2021;398(10294):27–40. 34102137 10.1016/S0140-6736(21)00797-2 PMC8436782

[27] Kang YK, Chen LT, Ryu MH, Oh DY, Oh SC, Chung HC, et al. Nivolumab plus chemotherapy versus placebo plus chemotherapy in patients with HER2-negative, untreated, unresectable advanced or recurrent gastric or gastro-oesophageal junction cancer (ATTRACTION-4): a randomised, multicentre, double-blind, placebo-controlled, phase 3 trial. Lancet Oncol. 2022;23(2):234–47. 35030335 10.1016/S1470-2045(21)00692-6

[28] Xu J, Jiang H, Pan Y, Gu K, Cang S, Han L, et al. Sintilimab plus chemotherapy for unresectable gastric or gastroesophageal junction cancer: the ORIENT-16 randomized clinical trial. JAMA. 2023;330(21):2064–74. 38051328 10.1001/jama.2023.19918 PMC10698618

[29] Yuan SQ, Nie RC, Jin Y, Liang CC, Li YF, Jian R, et al. Perioperative toripalimab and chemotherapy in locally advanced gastric or gastro-esophageal junction cancer: a randomized phase 2 trial. Nat Med. 2024;30(2):552–9. 38167937 10.1038/s41591-023-02721-w

[30] Li C, Tian Y, Zheng Y, Yuan F, Shi Z, Yang L, et al. Pathologic response of phase III study: perioperative camrelizumab plus rivoceranib and chemotherapy versus chemotherapy for locally advanced gastric cancer (DRAGON IV/CAP 05). J Clin Oncol. 2025;43(4):464–74. 39383487 10.1200/JCO.24.00795 PMC11776878

[31] Lin JX, Tang YH, Zheng HL, Ye K, Cai JC, Cai LS, et al. Neoadjuvant camrelizumab and apatinib combined with chemotherapy versus chemotherapy alone for locally advanced gastric cancer: a multicenter randomized phase 2 trial. Nat Commun. 2024;15(1):41. 38167806 10.1038/s41467-023-44309-5 PMC10762218

[32] Jiang H, Yu X, Li N, Kong M, Ma Z, Zhou D, et al. Efficacy and safety of neoadjuvant sintilimab, oxaliplatin and capecitabine in patients with locally advanced, resectable gastric or gastroesophageal junction adenocarcinoma: early results of a phase 2 study. J Immunother Cancer. 2022. 10.1136/jitc-2021-003635. 35296556 10.1136/jitc-2021-003635 PMC8928365

[33] Wei J, Lu X, Liu Q, Fu Y, Liu S, Zhao Y, et al. Neoadjuvant sintilimab in combination with concurrent chemoradiotherapy for locally advanced gastric or gastroesophageal junction adenocarcinoma: a single-arm phase 2 trial. Nat Commun. 2023;14(1):4904. 37580320 10.1038/s41467-023-40480-x PMC10425436

[34] Zhao Y, Li D, Zhuang J, Li Z, Xia Q, Li Z, et al. Comprehensive multi-omics analysis of resectable locally advanced gastric cancer: assessing response to neoadjuvant camrelizumab and chemotherapy in a single-center, open-label, single-arm phase II trial. Clin Transl Med. 2024;14(5):e1674. 38685486 10.1002/ctm2.1674 PMC11058238

[35] Janjigian YY, Al-Batran SE, Wainberg ZA, Muro K, Molena D, Van Cutsem E, et al. Perioperative durvalumab in gastric and gastroesophageal junction cancer. N Engl J Med. 2025;393(3):217–30. 40454643 10.1056/NEJMoa2503701

[36] Shitara K, Van Cutsem E, Bang YJ, Fuchs C, Wyrwicz L, Lee KW, et al. Efficacy and safety of pembrolizumab or pembrolizumab plus chemotherapy vs chemotherapy alone for patients with first-line, advanced gastric cancer: the KEYNOTE-062 phase 3 randomized clinical trial. JAMA Oncol. 2020;6(10):1571–80. 32880601 10.1001/jamaoncol.2020.3370 PMC7489405

[37] Zhang X, Wang J, Wang G, Zhang Y, Fan Q, Lu C, et al. First-line sugemalimab plus chemotherapy for advanced gastric cancer: the GEMSTONE-303 randomized clinical trial. JAMA. 2025;333(15):1305–14. 39992668 10.1001/jama.2024.28463 PMC11851304

[38] Johnson DB, Nebhan CA, Moslehi JJ, Balko JM. Immune-checkpoint inhibitors: long-term implications of toxicity. Nat Rev Clin Oncol. 2022;19(4):254–67. 35082367 10.1038/s41571-022-00600-w PMC8790946

[39] Tang Z, Wang Y, Liu D, Wang X, Xu C, Yu Y, et al. The Neo-PLANET phase II trial of neoadjuvant camrelizumab plus concurrent chemoradiotherapy in locally advanced adenocarcinoma of stomach or gastroesophageal junction. Nat Commun. 2022;13(1):6807. 36357415 10.1038/s41467-022-34403-5 PMC9649722

[40] Sullivan RJ, Weber JS. Immune-related toxicities of checkpoint inhibitors: mechanisms and mitigation strategies. Nat Rev Drug Discov. 2022;21(7):495–508. 34316029 10.1038/s41573-021-00259-5

[41] Zhang S, Chen X, Jin E, Wang A, Chen T, Zhang X, et al. The GSA family in 2025: a broadened sharing platform for multi-omics and multimodal data. Genomics Proteomics Bioinformatics. 2025;23(4):qzaf072. 40857552 10.1093/gpbjnl/qzaf072 PMC12451262

[42] CNCB-NGDC Members and Partners. Database resources of the National Genomics Data Center, China National Center for Bioinformation in 2025. Nucleic Acids Res. 2025;53(D1):D30–44. 39530327 10.1093/nar/gkae978 PMC11701749

