# Supplementary material for: Neoadjuvant immunochemotherapy plus thymalfasin in locally advanced gastric cancer: a prospective clinical trial
Source: BMC Med. 2026 Feb 26;24:143. doi: 10.1186/s12916-026-04740-z (PMC12964648; doi:10.1186/s12916-026-04740-z)
Supplement: Supplementary file 1 — Additional file 1. Study protocol. [file 12916_2026_4740_MOESM1_ESM.docx]

**A Prospective, Open-label, Single-arm Phase II Clinical Study to Investigate the Efficacy and Safety of PD-1 Monoclonal Antibody in Combination with Thymalfasin and SOX Neoadjuvant Therapy in the Treatment of cStage III Gastric/Gastroesophageal Junction Adenocarcinoma**

| **Protocol No.:** | **GATES** |
| --- | --- |
| **Version No.:** | **V1.0** |
| **Version Date:** | **30 November 2023** |
| **Principal Investigator:** | **Zekuan Xu** |
| **Study Site:** | **First Affiliated Hospital of Nanjing Medical University** |

**TABLE OF CONTENTS**

TABLE OF CONTENTS

[Protocol Signature Page 4](#_Toc10610)

[Protocol Synopsis 5](#_Toc32657)

[Study Flow Chart 9](#_Toc23531)

[1. Background 14](#_Toc6058)

[2. Objectives and Endpoints 16](#_Toc27447)

[2.1 Objectives 16](#_Toc12449)

[2.2 Endpoints 16](#_Toc25313)

[3. Study Design 17](#_Toc15513)

[4. Selection Criteria for Subjects 20](#_Toc13881)

[5. Study Termination and Withdrawal 23](#_Toc27762)

[5.1 Termination Criteria 23](#_Toc31273)

[5.2 Withdrawal Criteria 23](#_Toc13011)

[6. Study Process 24](#_Toc19092)

[6.1 Screening Period (≤28 days) 24](#_Toc18562)

[6.2 Neoadjuvant Therapy Period 24](#_Toc8151)

[6.3 Postoperative Follow-up Period 25](#_Toc1132)

[7. Use of Study Drugs 25](#_Toc15651)

[7.1 Serplulimab 25](#_Toc11535)

[7.2 Thymalfasin 26](#_Toc26307)

[7.3 Oxaliplatin and Tegafur 26](#_Toc22060)

[8. Dose Adjustments 26](#_Toc18325)

[8.1 General Principles 26](#_Toc13531)

[8.2 Doe Adjustment of Serplulimab 27](#_Toc26425)

[8.3 Management of Infusion-Related Reactions Associated with Serplulimab 28](#_Toc25982)

[8.4 Other Approved Dose Adjustments of Serplulimab 29](#_Toc24259)

[8.5 Dose Adjustment of Tegafur and Oxaliplatin 29](#_Toc14893)

[8.6 Principles for Managing Toxicity of Immune Checkpoint Inhibitor 31](#_Toc10226)

[8.7 Concomitant Medications and Concomitant Therapies 32](#_Toc7560)

[9. Management of Study Drugs 33](#_Toc832)

[9.1 Storage and Management of Study Drugs 33](#_Toc13592)

[9.2 Drug Recovery and Destruction 33](#_Toc4931)

[9.3 Records of Study Drugs 33](#_Toc1948)

[10. Efficacy Evaluation 33](#_Toc50)

[11. Safety Reports and Adverse Event Management 34](#_Toc20223)

[11.1 Definition of Adverse Event 34](#_Toc19605)

[11.2 Definition of Serious Adverse Events 34](#_Toc6863)

[11.3 Definition of Adverse Event 35](#_Toc3169)

[11.4 Recording of Adverse Events 37](#_Toc20313)

[11.5 Expedited Reporting of SAEs and Pregnancy 39](#_Toc31253)

[11.6 Immune-Related Adverse Events 39](#_Toc12007)

[12. Statistical Approach 39](#_Toc398)

[12.1 Statistical Analysis Data Set 40](#_Toc17842)

[12.2 Statistical Analysis Plan 40](#_Toc22189)

[12.3 Efficacy Analysis 40](#_Toc5755)

[12.4 Drug Safety Evaluation 40](#_Toc7686)

[13. Quality Control and Quality Assurance 40](#_Toc5362)

[14. Ethical, Regulatory, and Administrative Principles 41](#_Toc8242)

[14.1 Ethical Principles 41](#_Toc9301)

[14.2 Laws and Regulations 41](#_Toc1143)

[14.3 Data Protection 41](#_Toc17378)

[14.4 Confidentiality Agreement 41](#_Toc13791)

[14.5 Record Safekeeping 41](#_Toc8219)

[14.6 Early Suspension of Study 41](#_Toc28161)

[14.7 Sponsor's Audits and Regulatory Authority's Inspections 42](#_Toc14060)

[14.8 Protocol Amendments 42](#_Toc31490)

[14.9 Ownership and Use of Study Data and Results 42](#_Toc28207)

[14.10 Publication 42](#_Toc17040)

# Protocol Signature Page

I, as a participating doctor/statistician, have read the protocol of this study.

I have fully discussed the objectives of this study and the contents of this protocol with the study director.

I agree to conduct the study in accordance with this protocol, protocol requirements, ethic principles, and under the guidance of Good Clinical Practice (GCP).

I agree that the contents of this protocol will be kept confidential, will not be disclosed to third parties and will only be used for the conduct of this study.

I understand that I will be notified in writing if this study is terminated prematurely or suspended at any time for whatever reason. Similarly, if I decide to withdraw from the conduct of this study, I will immediately notify the leading site and the principal investigators of the study in writing.

Unit: ________________

Signature: ________________

Date: ________________

# Protocol Synopsis

| **Study Title** | A Prospective, Open-label, Single-arm Phase II Clinical Study to Investigate the Efficacy and Safety of PD-1 Monoclonal Antibody in Combination with Thymalfasin and SOX Neoadjuvant Therapy in the Treatment of cStage III Gastric/Gastroesophageal Junction Adenocarcinoma |
| --- | --- |
| **Sponsor** | The First Affiliated Hospital of Nanjing Medical University (Jiangsu Province Hospital) |
| **Study Drugs** | Serplulimab, thymalfasin |
| **Study Phase** | Phase II |
| **Objectives** | To evaluate the efficacy and safety of Serplulimab in combination with thymalfasin and SOX regimen for neoadjuvant therapy of locally advanced gastric cancer |
| Endpoints | Primary endpoint:   - Pathologic complete response (pCR) rate;   Secondary endpoints:   - Clinical downstaging rate (T and/or N downstaging) after neoadjuvant therapy; - Tumor regression grade (TRG) after neoadjuvant therapy; - R0 resection rate after neoadjuvant therapy; - Major pathologic response (MPR) rate after neoadjuvant therapy; - Objective response rate (ORR) and disease control rate (DCR) after neoadjuvant therapy (RECIST v1.1); - Disease-free survival (DFS) and overall survival (OS) after neoadjuvant therapy; - Safety of neoadjuvant therapy (adverse reactions caused by drug treatment, perioperative complications, etc.).   Exploratory endpoint:   - Expression of CD68, CD86, CD163, CD4^+^ T cells, and CD8^+^ T cells in tumor tissue specimens. |
| **Study Design** | This is a prospective, single-center, single-arm, phase II clinical study. This study plans to enroll 30 treatment-naïve and operable patients with locally advanced adenocarcinoma gastric. After signing the informed consent form, patients will be screened to ensure they meet the inclusion and exclusion criteria. Before surgery, patients will receive three cycles of neoadjuvant therapy with Serplulimab combined with the SOX regimen and nine weeks of thymalfasin. The efficacy of neoadjuvant therapy and the feasibility of radical D2 resection will be assessed via imaging after Cycle 2 and Cycle 3. Radical gastrectomy will be performed within 2–6 weeks after completing the third dose. The postoperative treatment of patients will be jointly decided by clinical physicians and patients according to the actual conditions of clinical diagnosis and treatment.  The main observation indicator is the pCR rate after neoadjuvant therapy;  Safety assessment: The safety will be assessed once after each cycle of neoadjuvant therapy and at 30 days after surgery;  Event follow-up: The events will be followed up every 3 months during the first year after surgery, and once every 6 months from the first to the second years, until the second year. |
| **Inclusion Criteria** | Patients eligible for enrollment in this study must meet all of the following criteria:   1. Male or female patients aged 18–75 years with G/GEJ adenocarcinoma; 2. According to the AJCC 8th edition gastric cancer staging, patients with cStage III (cT_3-4a_N_1-3_M_0_) assessed by abdominal CT and G/GEJ adenocarcinoma diagnosed by gastroscopy and pathology (HER-2 negative or 1+); and patients with gastroesophageal junction (GEJ) cancer will only allow Siewert type III, and Siewert type II subjects who do not require combined thoracotomy to be enrolled. 3. Before enrollment, a doctor in charge of gastrointestinal surgery and an imaging technician jointly assess that the tumor is cStage III and qualified for the study of R0 resection with the purpose of cure, and the patient agrees to receive radical surgery and has no contraindication to surgery as judged by the surgeon; 4. No previous systemic therapy for the current disease, including surgical treatment, anti-tumor chemoradiotherapy/immunotherapy; 5. Expected survival of ≥3 months; 6. There are measurable tumor lesions according to RECISTv1.1 (see Attachment 3 for details); 7. ECOG PS score (see Attachment 4 for details) of 0–1 within 7 days after the first dose; 8. The heart is functioning well and resection for curative purposes can be performed. Patients with underlying ischaemic, valvular or other significant cardiac disease should be evaluated preoperatively by a cardiologist, if clinically indicated; 9. Primary organs are functioning normally and subjects are required to meet the following laboratory parameters:    1. Absolute neutrophil count (ANC) ≥ 1.5 × 10^9^/L in the absence of granulocyte colony-stimulating factor in the past 14 days;    2. Platelets ≥ 100 × 10^9^/L without transfusion in the past 14 days;    3. Hemoglobin > 9 g/dL without or erythropoietin use within the past 14 days;    4. Total bilirubin ≤ 1.5 × upper limit of normal (ULN); if total bilirubin > 1.5 × ULN but direct bilirubin ≤ ULN, the patient is also allowed to be enrolled;    5. Aspartate aminotransferase (AST) and alanine aminotransferase (ALT) ≤ 2.5 × ULN    6. Serum creatinine ≤ 1.5 × ULN and creatinine clearance (calculated using the Cockcroft-Gault formula) ≥ 60 ml/min;    7. Good coagulation, defined as international normalized ratio (INR) or prothrombin time (PT) ≤ 1.5 times ULN;    8. Normal thyroid function, defined as thyroid stimulating hormone (TSH) within the normal range. If baseline TSH is outside the normal range, subjects with total T3 (or FT3) and FT4 within the normal range can also be enrolled;    9. Myocardial enzymes within the normal range (subjects with isolated laboratory abnormalities judged as not clinically significant by the investigator are also allowed to be enrolled); 10. Thyroid function indicators: thyroid stimulating hormone (TSH) and free thyroxine (FT3/FT4) within the normal range or with mild and clinically insignificant abnormalities; 11. Body weight above 40 kg (including 40 kg), or BMI > 18.5; 12. Female patients must meet:  - Patients with menopausal (defined as no menses for at least 1 year and no other confirmed cause other than menopause) status, or who have been surgically sterilized (removal of ovaries and/or uterus), or who are of childbearing potential must also meet the following requirements: - Pregnancy test must be negative within 7 days prior to first dose; - Agree to use contraception with an annual failure rate of < 1% or remain abstinent (avoid heterosexual intercourse) from signing of informed consent through at least 120 days after the last dose of study drug and for at least 9 months after surgery (contraceptive methods with an annual failure rate of < 1% include bilateral tubal ligation, male sterilization, correct use of hormonal contraceptives that can inhibit ovulation, hormone-releasing intrauterine devices, and copper-containing intrauterine devices.) ; - Do not breastfeed.  1. Male patients must meet:   Agree to practice abstinence (avoid heterosexual intercourse) or use contraception as defined below: Male patients must remain abstinent or use condoms correctly for at least 120 days after the last dose of study drug and for at least 9 months after surgery if the partner is a woman of childbearing potential or if the partner is pregnant. Reliability of sexual abstinence should be evaluated with reference to the duration of the clinical study, patient preferences, and lifestyle of daily living. Periodic abstinence (eg, calendar day, ovulation, basal body temperature, or post-ovulation contraceptive methods) and withdrawal are not acceptable methods of contraception;   1. Subjects read and fully understood the patient information and signed the informed consent form. |
| **Exclusion Criteria** | Subjects who meet any of the following criteria will not be enrolled in this study:   1. Patients with prior (within 5 years) or concurrent other malignancy. Patients with cured localized tumors, such as cutaneous basal cell carcinoma, cutaneous squamous cell carcinoma, superficial bladder cancer, prostate carcinoma in situ, cervical carcinoma in situ, breast carcinoma in situ, stage I lung cancer, and stage I colorectal cancer, can be enrolled; 2. Patients who are scheduled to undergo or have previously undergone organ or bone marrow transplant; 3. Patients who have received blood transfusion within 2 weeks before the first dose or have a history of bleeding, and experience any bleeding event with a severity of grade 3 or higher based on CTCAE 4.0 within 4 weeks before screening; 4. Patients with abnormal coagulation function and bleeding tendency (INR > 1.5 in the absence of anticoagulants); patients treated with anticoagulants or vitamin K antagonists such as warfarin, heparin or their analogues; patients treated with low-dose warfarin (1 mg orally once daily) or low-dose aspirin (no more than 100 mg daily) for preventive purposes are allowed under the premise of prothrombin time international normalized ratio (INR) ≤ 1.5; 5. Have a history of arterial/venous thrombotic events within 6 months before screening, such as cerebrovascular accident (including transient ischemic attack), deep venous thrombosis (venous thrombosis caused by venous catheterization for previous chemotherapy is excluded if the investigator judges that the patient has recovered) and pulmonary embolism; 6. Have a history of myocardial infarction and poorly controlled arrhythmia within 6 months before the first dose (including QTc interval ≥ 450 ms for males and ≥ 470 ms for females) (QTc interval are calculated with Fridericia formula); 7. Presence of NYHA criteria class III-IV cardiac dysfunction or echocardiography: LVEF (left ventricular ejection fraction) < 50%; 8. Urine routine suggests urine protein ≥ + + and confirms 24-hour urine protein > 1.0 g; 9. There are multiple factors affecting oral medication (such as inability to swallow, chronic diarrhea and intestinal obstruction, etc.); 10. Pleural or peritoneal effusion with clinical symptoms requiring clinical intervention; 11. Human immunodeficiency virus (HIV) infection; 12. Active pulmonary tuberculosis; 13. Chronic unhealed wounds or incompletely healed fractures; 14. Patients with previous and current interstitial pneumonia, pneumoconiosis, radiation pneumonitis, drug-related pneumonia, severely impaired pulmonary function, etc. that may interfere with the detection and treatment of suspected drug-related pulmonary toxicity; 15. Presence of known active or suspected autoimmune disease, except for those who are in a stable state at enrollment (not requiring systemic immunosuppressive therapy); 16. History of severe chronic autoimmune diseases, such as systemic lupus erythematosus; history of inflammatory bowel diseases such as ulcerative enteritis and Crohn 's disease; history of chronic diarrheal diseases such as irritable bowel syndrome; history of sarcoidosis or tuberculosis; history of active hepatitis B and C and HIV-infected patients; patients with well-controlled non-serious immune diseases, such as dermatitis, arthritis, psoriasis, etc., can be enrolled. Patients with hepatitis B virus titer < 500 copies/ml can be enrolled; 17. Patients requiring treatment with systemic corticosteroids (at dose level > 10 mg/day prednisone efficacy) or other immunosuppressive drugs within 14 days prior to the first dose or during the study. However, enrollment is permitted if: In the absence of active autoimmune disease, patients are permitted to use topical or inhaled steroids, or adrenal hormone replacement therapy at dose level ≤ 10 mg/day prednisone efficacy; 18. Any active infection requiring systemic anti-infective treatment within 14 days before the first dose, with the exception of prophylactic antibiotic therapy (eg, prevention of urinary tract infection or chronic obstructive pulmonary disease); 19. Treatment with live vaccines within 28 days prior to the first dose; except for inactivated viral vaccines for seasonal influenza; 20. Prior treatment with antibody/drug therapy against immune checkpoints, such as PD-1, PD-L1, CTLA-4 inhibitors; 21. Treatment with related drugs or medical technology affecting immunity within 6 months prior to the first dose (including but not limited to: thymopentin, thymalfasin, interferon, CAR-T therapy, etc.); 22. Patients who are receiving other clinical study treatment, or planning to start this study treatment less than 1 month before the end of previous clinical study treatment; 23. Known history of allergy or intolerance to any study medications or their components; 24. Patients with a history of alcohol abuse, drug abuse and drug abuse. Patients who have stopped drinking alcohol can be enrolled; 25. Patients who do not follow the doctor 's advice, do not take medicine according to the regulations, or have incomplete data that may affect the efficacy judgment or safety judgment; 26. Pregnant or lactating female patients; 27. Patients with conditions that may increase the risk of study participation and study medications, or other severe, acute, or chronic diseases that, in the judgment of the investigator, will make them inappropriate for participation in a clinical study. 28. Patients with other conditions unsuitable for this clinical trial judged by the investigator. |
| Administration Method of the Study Drugs | Neoadjuvant therapy phase:  Serplulimab: 300 mg, i.v., D1, Q3W;  Thymalfasin: 4.8 mg, subcutaneous injection, twice a week. On Day 1 and Day 4 of each week during Weeks 1–9 or until surgery;  Oxaliplatin: 130 mg/m2, i.v., D1, Q3W;  Tegafur: oral administration: body surface area < 1.25 m^2^, 40 mg each time; body surface area ≥ 1.25 to < 1.5 m^2^, 50 mg each time; body surface area ≥ 1.5 m^2^, 60 mg each time, twice daily for each treatment cycle at D1-D14  After 3 courses of treatment before surgery, radical gastrectomy will be conducted based on evaluation by the investigator within 2–6 weeks after completion of chemotherapy + immunotherapy.  Adjuvant therapy phase: The postoperative treatment of patients will be jointly decided by clinical physicians and patients according to the actual conditions of clinical diagnosis and treatment. |
| **Evaluation Criteria** | Efficacy evaluation:  Evaluation per RECIST v1.1 criteria:   - Pathologic complete response (pCR) rate, defined as the proportion of subjects with no residual viable tumor cells under microscopic examination and negative lymph nodes among the total subjects; - Major pathologic response (MPR) rate, defined as the proportion of subjects with ≤10% viable tumor cells in the resected specimen among the total subjects; - Tumor regression grade (TRG) - Objective response rate (ORR), defined as the proportion of subjects achieving complete response (CR) and partial response (PR) among the total subjects; - Clinical downstaging rate: ypT0 ratio, ypN0 ratio, and preoperative imaging clinical stage will be counted, respectively, to compare with baseline imaging clinical stage downstaging ratio. - Disease-free survival (DFS), defined as the time from surgery to the first radiological disease recurrence or death (whichever occurs first); - Overall survival (OS), defined as the time from enrollment to death of the subject due to any cause;   Safety evaluation:   - Drug-related safety (per CTCAE 5.0): incidence, relationship to study drug and severity of TRAEs, TRAEs ≥ Grade 3, irAEs, etc. - Surgery-related safety (according to Dindo-Demartines-Clavien classification). |
| **Statistical Protocol** | General statistical analysis methods  All statistical analyses will be programmed and calculated using SPSS15 Statistical Analysis Software. Intergroup comparisons will be presented with 95% confidence intervals and p-values.  Unless otherwise specified, measurement data will be statistically described using mean ± standard deviation or median (minimum, maximum). Enumeration data will be statistically described using frequencies (percentages). |

**Study Flow Chart**

| **Study Phase** | **Screening period** | | **Treatment period (every 3 weeks (21 days) as one treatment cycle)** | | | **Study treatment discontinuation** | **Follow-up period** |
| --- | --- | --- | --- | --- | --- | --- | --- |
|  |  |  | **Neoadjuvant therapy** | | **Surgery** |  |  |
| **Treatment cycle/visit name** | Screening period | | Cycle 1 | Cycles 2–3 | Surgery | Study treatment discontinuation | Follow-up of events |
| **Visit time** |  | |  | |  | Becoming aware of or confirming the discontinuation of study treatment | 1 year after surgery: every 3 months  1–2 years after surgery: every 6 months |
| **Window period (days)^1^** | -28 to -8 | -7 to -1 |  | ±3 | ±7 | ±7 | 1 year after surgery: ±7  1–2 years after surgery: ±15 |
| Informed consent form | **X** | |  |  |  |  |  |
| Inclusion and exclusion criteria | **X** | |  |  |  |  |  |
| Demographics and medical history | **X** | |  |  |  |  |  |
| Prior treatment and concomitant treatment**^2^** | **X** | |  | **X** | **X** | **X** |  |
| Height, weight, and vital signs**^3^** | **X** | |  |  |  |  |  |
| Adverse events**^4^** | **X** | | **X** | **X** | **X** | **X** |  |
| Echocardiography | **X** | |  |  |  |  |  |
| 12-lead electrocardiogram | **X** | |  |  | **X** |  |  |
| Complete physical examination | **X** | |  |  |  |  |  |
| Symptom-directed physical examination | **X** | | **X** | **X** | **X** | **X** |  |
| ECOG score |  | **X** | **X** | **X** | **X** | **X** |  |
| Gastroscopy**^5^** | **X** | |  |  |  |  |  |
| PD-L1 expression (CPS score)**^6^** | **X** | |  |  |  |  |  |
| MSI status**^7^** | **X** | |  |  |  |  |  |
| Subsequent anti-tumor treatment |  | |  |  |  |  | **X** |
| Survival status |  | | **X** | **X** | **X** | **X** | **X** |
| Serplulimab**^8^** |  | | **X** | **X** |  |  |  |
| Oxaliplatin + Tegafur (SOX)**^9^** |  | | **X** | **X** |  |  |  |
| Thymalfasin**^10^** |  | | **X** | **X** |  |  |  |
| Hematology, biochemistry, coagulation, urinalysis**^11^** |  | **X** | **X** | **X** | **X** |  |  |
| Thyroid function**^12^** |  | **X** | **X** | **X** | **X** |  |  |
| Cardiac function markers**^13^** |  | **X** | **X** | **X** | **X** |  |  |
| Tumor markers**^14^** |  | **X** | **X** | **X** | **X** |  |  |
| Pre-transfusion eight-item panel**^15^** |  | **X** |  |  |  |  |  |
| Postoperative pathological examination |  | |  |  | **X** |  |  |
| Imaging examination**^16^** | **X** | |  | **X** | **X** |  | **X** |
| Tumor tissue sample**^17^** | **X** | |  |  | **X** |  |  |
| **Notes:**   1. The screening period has a window of 28 days, the treatment period has a window of ±3 days (with a tumor assessment window of ±7 days), the end-of-treatment visit has a window of ±7 days, and the follow-up period has a window of ±7 days. During the screening period, ECOG performance status, hematology, blood biochemistry, coagulation, urinalysis, T3 or FT3, FT4, and TSH must be recorded within 7 days prior to the first dose and meet the corresponding inclusion and exclusion criteria for enrollment. 2. All prior treatments within 30 days before signing the ICF and all concomitant treatments from the signing of the ICF until the discontinuation of study treatment will be documented; thereafter, concomitant treatments for SAEs related to the study drug need to be documented. 3. Height examination is only performed in the screening period; vital signs include body temperature, pulse, respiratory rate and blood pressure. Body weight will be measured prior to each dose. If the subject's weight during the study changes by ≤10% compared to the baseline reference weight, no dose adjustment of the study drug is required. If the weight change exceeds 10%, the dose needs to be recalculated. 4. AEs and SAEs should be recorded from the signing of the ICF until 30 days after the last dose of the study drug. 5. Gastroscopy and pathology tests: Subjects must complete gastroscopy and pathology tests during the screening period after meeting the enrollment criteria and signing the informed consent form to determine the distance between the tumor and the incisors and collect tumor tissue specimens for subsequent laboratory tests. If gastroscopy and pathology tests have been performed at an external institution, gastroscopy must be repeated at this study site, but there are no requirements for pathology tests. 6. PD-L1 expression of gastric cancer: Subjects must complete the immunohistochemical PD-L1 expression and CPS scoring of gastric cancer tissues during the screening period after meeting the inclusion criteria and signing the informed consent form. They need to be completed at this study site. 7. MSI status of gastric cancer: Subjects must complete the immunohistochemical MSI status testing of gastric cancer tissues during the screening period after meeting the inclusion criteria and signing the informed consent form. They need to be completed at this study site. 8. Serplulimab will be administered on Day 1 of each 3-week treatment cycle after completion of all clinical and laboratory procedures/assessments. During the neoadjuvant phase, treatment consists of 3 cycles with a dose volume of 300 mg/dose. The dosing window is within ±3 days of the scheduled time point. 9. After completion of all clinical and laboratory procedures/evaluations, neoadjuvant therapy will be administered using the standard SOX regimen (oxaliplatin + Tegafur), with 3 weeks as one treatment cycle, and neoadjuvant therapy consists of a total of 3 cycles. Oxaliplatin will be administered on Day 1 of each cycle at a dose of 130 mg/m^2^, via intravenous infusion over 2–6 hours (chemotherapy drugs will be administered after the completion of Serplulimab infusion). If the body surface area exceeds 2.0 m^2^, the dose is calculated based on 2.0 m^2^. Tegafur will be administered orally according to the body surface area as below: <1.25 m^2^, 40 mg/dose; ≥1.25 to <1.5 m^2^, 50 mg/dose; ≥1.5 m^2^, 60 mg/dose; twice daily, from Day 1 to Day 14 of each treatment cycle. 10. After completion of all clinical and laboratory procedures/assessments, thymalfasin 4.8 mg will be administered via subcutaneous injection, twice weekly starting from Day 1 of neoadjuvant therapy. On Day 1 and Day 4 of each week during Weeks 1–9. 11. Routine laboratory tests include hematology, blood biochemistry, urinalysis, and coagulation function. The hematology items include complete blood count with differential. The blood biochemistry items include blood urea/urea nitrogen, creatinine, sodium, potassium, magnesium, chloride, bicarbonate/carbon dioxide combining power/total carbon dioxide (TCO2), calcium, phosphorus, glucose, total bilirubin, direct bilirubin, alanine aminotransferase (ALT), aspartate aminotransferase (AST), alkaline phosphatase, lactate dehydrogenase, total cholesterol, total protein, and albumin. The urinalysis items include specific gravity, pH, glucose, protein, casts, ketones, and blood cells. Coagulation function tests include activated partial thromboplastin time (APTT), prothrombin time (PT), and international normalized ratio (INR). The tests should be performed within 7 days before the first dose, within 3 days before dosing in Cycle 3, within 7 days before surgery, and within 3–7 days after surgery. If the laboratory tests and study drug administration are scheduled on the same day, the study drug cannot be administered until the test results are obtained. The analysis will be conducted in this study site. 12. Thyroid function tests include analysis of triiodothyronine (T3 or FT3), thyroxine (T4 or FT4), and thyroid-stimulating hormone (TSH). The tests should be performed 7 days before the first dose, 3 days before dosing in Cycle 3, 7 days before surgery, and within 3–7 days after surgery. The analysis will be conducted in this study site. 13. Cardiac function markers include: myocardial enzymes [creatine kinase (CK) and its isoenzymes], troponin (TnT or TnI), and brain natriuretic peptide (BNP and/or NT-proBNP). The tests should be performed 7 days before the first dose, 3 days before dosing in Cycle 3, 7 days before surgery, and within 3–7 days after surgery. The analysis will be conducted in this study site. 14. Tumor markers must include: carcinoembryonic antigen (CEA), carbohydrate antigen CA19-9, and alpha-fetoprotein (AFP). The tests should be performed 7 days before the first dose, 3 days before dosing in Cycle 3, 7 days before surgery, and within 3–7 days after surgery. The analysis will be conducted in this study site. 15. Pre-transfusion eight-item panel: All subjects must undergo hepatitis B surface antigen (HBsAg) and hepatitis B core antibody (HBcAb) testing during the screening period. For subjects with HBsAg (+) or HBcAb (+), further hepatitis B virus (HBV) DNA titer testing is required. At screening (baseline), in case of HBV DNA (-), and either 1. HBsAg (+), or 2. HBcAb (+), HBsAg (-), then HBV surface antigen/core antibody and HBV DNA must be tested every 2 cycles during treatment, before the surgery, at treatment discontinuation, and during safety follow-up. 16. CT or MRI imaging examinations should be performed within the screening period, within 7 days before treatment in Cycle 3 of neoadjuvant therapy, within 1 week after completion of neoadjuvant therapy, and after the surgery until the initiation of adjuvant therapy (recommended 4–12 weeks after the surgery). Throughout the study period, the examination methods should remain as consistent as possible. Abdominal CT scans require dual-phase (arterial and venous) imaging, performed under fasting conditions with the intake of at least 1000 mL of water or contrast agent. Contrast agents should be used unless contraindicated. The investigator assesses tumor imaging findings according to RECIST v1.1 (the frequency of tumor assessments may be increased as clinically needed at the investigator's discretion). The investigator should determine subsequent treatment based on the efficacy evaluation results. If tumor assessment has been performed within 28 days prior to the first dose, and the same method and equipment are used at the same hospital, it may serve as the baseline tumor assessment. 17. Subjects must provide formalin-fixed, paraffin-embedded (FFPE) tumor samples (paraffin blocks or unstained sections) collected from the lesion site at or after GC diagnosis (preferably within 2 months before the first dose of the study drug), along with relevant pathological reports of the aforementioned specimens. If the subject does not have archived tumor tissue samples that meet the requirements, a fresh tumor lesion biopsy must be performed during the screening period to obtain corresponding tumor samples (the number of specimens collected will be determined based on the biopsy). Additionally, surgically excised tumor tissue specimens will be retained postoperatively. Tumor tissue section samples will be used for determination of PD-L1 expression levels and MSI testing. | | | | | | | |

1. **Background**

Gastric cancer (GC) is a primary malignant tumor of epithelial origin in the stomach. Among malignant tumors worldwide, gastric cancer has the third highest mortality rate, and in China, the incidence of gastric cancer ranks second only to lung cancer, and the mortality rate ranks third. In the world, there are about 1.2 million new cases of gastric cancer each year, and China accounts for about 40% of them. The proportion of early-stage gastric cancer in China is very low, only about 20%. Most cases are already in the advanced stage when they are detected, with a proportion as high as 50%–60%, and the overall 5-year survival rate is less than 50%. At present, radical surgery is still the only way to cure gastric cancer, but the surgical resection rate is low, and the R0 resection rate is about 70–80%; patients with stage II or higher have a high postoperative recurrence rate. Therefore, increasing the surgical resection rate and seeking other treatments that are more effective other than surgical treatment are the development direction of gastric cancer treatment research.

Neoadjuvant therapy can reduce tumor stage and increase the likelihood of complete tumor resection to achieve maximum pathological response. Today, the role of neoadjuvant therapy in potentially resectable gastric cancer is gradually being recognized. The results of a large multicenter MAGIC study from Europe showed that patients with potentially resectable gastric cancer who underwent surgery after neoadjuvant chemotherapy had a higher R0 resection rate (79.3% vs. 70.3%, P < 0.05), overall survival, progression-free survival, and 5-year survival rate (36% vs. 23%, P < 0.05) compared with patients who underwent surgery alone. The results of the FNCLCC/FFCD phase III clinical trial also showed that patients with resectable gastric cancer who underwent surgery after neoadjuvant chemotherapy were significantly better than those in the surgery alone group in terms of R0 resection rate (84% vs. 73%, P = 0.04), overall survival (P = 0.02), progression-free survival (P = 0.003), and 5-year survival rate (38% vs. 24%, P = 0.02). Both studies demonstrated the efficacy and safety of neoadjuvant chemotherapy for gastric cancer, and served as the key evidence for the inclusion of neoadjuvant chemotherapy in combination with surgical treatment in the NCCN guidelines for the diagnosis and treatment of gastric cancer.

In May 2017, the FDA approved pembrolizumab for the treatment of advanced NSCLC based on a non-squamous NSCLC population with high PD-L1 (programmed death ligand 1) expression, ushering a new era for the treatment of tumors with immune checkpoint inhibitors (ICIs). Immune checkpoint inhibitors activate T cells by blocking the co-inhibitory signals of two pairs of immune checkpoints, CTLA-4/B7-1 and PD-1/PD-L1, thereby achieving anti-tumor immunotherapy. In recent years, there are frequent good results in the exploration of immunotherapy combined with chemotherapy in the treatment of gastric cancer. ATTRCTION-04 was a randomized, double-blind, Phase III study in Asian patients with gastric cancer that showed a significant increase in PFS, but no improvement in overall survival, when Nivo was combined with chemotherapy compared to placebo combined with chemotherapy in patients with HER2-negative advanced, unresectable gastric and gastroesophageal junction cancer (G/GEJ). Another Checkmate-649 study, which also used Nivo combined with chemotherapy, prolonged OS and PFS in the population of patients with CPS ≥ 5 compared with the chemotherapy alone group, establishing the position of immune combined chemotherapy in the first-line treatment of gastric cancer. In published data from another phase III randomized controlled study, ORIENT-16, chemotherapy (oxaliplatin + capecitabine) combined with immunotherapy (Serplulimab) significantly reduced the risk of death compared with chemotherapy alone in the overall population and in the population with PD-L1 CPS ≥ 5 (ie, risk reduction of 23.4% and 34.0%, respectively). In April 2021 and June 2022, Nivo and Sintilimab were successively approved by the National Medical Products Administration (NMPA) for first-line treatment in combination with chemotherapy in patients with locally advanced unresectable or metastatic gastric cancer, gastroesophageal junction (GEJ) cancer, and oesophageal adenocarcinoma.

PD-1 inhibitors have now achieved excellent results in the treatment of advanced G/GEJ adenocarcinoma. However, whether immunotherapy can play a better role in the earlier stage of gastric cancer treatment, such as neoadjuvant therapy, will be a research hotspot in the field of gastric cancer treatment in the future. At present, a number of clinical studies of PD-1 monoclonal antibody combined with chemotherapy as neoadjuvant therapy have been published at the ASCO 2022 GI meeting. For example: for NCT04890392, the conference reported that PD-1 combined with SOX (oxaliplatin + Tegafur) in the treatment of resectable locally advanced gastric/gastroesophageal junction adenocarcinoma achieved pCR in 23.8% of patients and MPR in 61.9% of patients. Another single-arm Phase II clinical study from China also demonstrated that the combination of PD-1 inhibitor with oxaliplatin/capecitabine in neoadjuvant therapy showed encouraging pCR rate and favorable safety. However, although immune neoadjuvant therapy for gastric cancer has achieved good results, according to data from multiple studies, its pCR rate remains only about 20%.

It is indicated in recent literature that immune checkpoint inhibitors act synergistically with thymalfasin [Thymosin α1 (Tα1)]. As shown in a paper published in 2015, thymalfasin administered at different doses had a tumor-shrinking effect on C57BL/6 mouse models of melanoma, demonstrating for the first time that thymalfasin combined with PD-1 monoclonal antibody can inhibit melanoma growth and lung metastasis in B6D2F1 mouse model. According to a retrospective clinical study in 2018, the sequential IPI monoclonal antibody of thymalfasin (CTLA-4 monoclonal antibody) for treating patients with metastatic melanoma can significantly increase the mOS to 38.4 months compared to only 8 months of patients untreated with thymalfasin (P = 0.03). Meanwhile, compared to those of patients untreated with thymalfasin, the 2-, 3-, 4- and 5-year OS rates of patients pretreated with thymalfasin were significantly increased, with statistical differences (P < 0.05). A preclinical study published in 2020 showed that in a mouse model of immune checkpoint inhibitor-induced colitis, thymalfasin can ameliorate the colonic histopathology, restore the integrity of epithelial barrier, and avoid the influence of gastrointestinal toxicity of immune checkpoint inhibitor by offsetting inflammatory pathology and providing mucosal homeostasis. An ongoing randomized phase II study in China aims to explore whether the addition of Tα1 to the protocol of PD-1 inhibitor combined with a vascular endothelial growth factor receptor (VEGFR) inhibitor can further improve ORR and progression-free survival (PFS) of patients with advanced gastric cancer who have failed at least two treatment protocols. In this study, Tα1 was subcutaneously injected at the dose of 4.8 mg twice a week, and preliminary analysis showed raised ORR, prolonged PFS, and favorable safety (data unpublished yet; ChiCTR1900025367).

Through reviewing its mechanism of action, thymalfasin is an immunological peptide with 28 amino acids, which has potential therapeutic effects on patients with various tumors (such as liver cancer, lung cancer, and lymphoma) and diseases (including infectious diseases and chronic hepatitis B), as well as immune improvement effects (*e.g.*, enhancing the response to vaccine) on patients with immunologic inadequacy. It mainly acts on Toll-like receptor 2 (TRL2) and TRL9 on dendritic cells (DCs), and stimulates intracellular signaling pathways including the NF-κB and p38 MAPK pathways, thus increasing the activity of natural killer (NK) cells. Moreover, it promotes the conversion of helper T (Th) cells to Th1 subset, increases the expression of Th1-type cytokines like IL-2 and IFN-γ, elevates the level of cytotoxic T lymphocytes (CTLs), and facilitates DC activation. Tumor cells can be killed through the combined action of NK cells, cluster of differentiation 4 (CD4)^+^ Th1 cells and CTLs. Meanwhile, activated DCs can also generate anti-tumor responses and perform efficient antigen presentation.

Based on the above background and basis, we designed this study to investigate the efficacy and safety of Serplulimab in combination with thymalfasin and SOX regimen for neoadjuvant therapy of locally advanced gastric cancer, and look forward to exploring safer and more effective neoadjuvant regimens for such patients to further benefit patients with gastric cancer.

1. **Objectives and Endpoints**
   1. **Objectives**
2. Primary objective: To investigate the efficacy and safety of Serplulimab in combination with thymalfasin and SOX regimen for neoadjuvant therapy of locally advanced gastric cancer.
3. Secondary objective: To investigate the effect of neoadjuvant therapy with Serplulimab in combination with thymalfasin and SOX regimen on tumor immune micro-environment in locally advanced gastric cancer.
   1. **Endpoints**
      1. **Primary Endpoint**

- Pathological Complete Response (pCR): For patients with surgically resectable gastric cancer after neoadjuvant therapy, the proportion of patients with no residual tumor cells in the primary site to the total enrolled patients.
  - 1. **Secondary Endpoints**
- Major Pathological Response (MPR): For patients with surgically resectable gastric cancer after neoadjuvant therapy, the proportion of patients with less than 10% residual tumor cells in the primary site to the total enrolled patients.
- Tumor Regression Grade (TRG): It is intended to grade the pathological response of tumor after neoadjuvant therapy and generally divide the grade mainly according to the proportion of fibrosis and residual tumor in the tumor. In this study, Becker criteria are used to grade TGR as follows: TRG1a (no residual tumor), equivalent to pCR; TRG1b (< 10% residual tumor); TRG2 (10% to 50% residual tumor); and TRG3 (> 50% residual tumor). For patients with surgically resectable gastric cancer after neoadjuvant therapy, the proportion of patients with less than 10% residual tumor cells in the primary site to the total enrolled patients (TRG1a & TRG1b).
- Clinical downstaging rate (T and/or N downstaging): ypT0 ratio, ypN0 ratio, and preoperative imaging clinical stage will be counted, respectively, to compare with baseline imaging clinical stage downstaging ratio.
- Objective response rate (ORR): It refers to the proportion of patients whose tumor shrinks to a certain extent and remains for a certain period of time, including CR (Complete Response) and PR (Partial Response) cases. Objective tumor response will be assessed using Response Evaluation Criteria in Solid Tumors (RECIST 1.1 criteria). Patients must have measurable tumor lesions at baseline. Response evaluation criteria are divided into Complete Response (CR), Partial Response (PR), Stable Disease (SD) and Progressive Disease (PD) according to RECIST 1.1 criteria.
- Disease control rate (DCR): It refers to the percentage of confirmed complete response (CR), partial response (PR) and stable disease (SD) cases among evaluable efficacy patients.
- Disease-free Survival (DFS): Disease-free survival refers to the time from the start of randomization to disease recurrence or death due to disease progression. DFS will be defined as the last date the patient is last confirmed to be disease-free survival if the patient does not experience disease progression during the study. Patients who discontinue the trial for reasons other than disease progression (no subsequent imaging) and patients who receive treatment after the trial will be censored at the time of discontinuation or the time of initiation treatment after the trial. When patients are not censored at the time of discontinuation from the trial or the time of initiation treatment after the trial, pre-planned sensitivity statistical analyses will further confirm DFS based only on the time of event of radiologically confirmed progression. New onset of other tumors are not considered as disease progression events and are not censored. If an imaging examination and evaluation show disease progression, the date of disease progression will not be the imaging examination date when disease progression is first shown, but the imaging examination date when disease progression is clearly confirmed. If disease progression is diagnosed by other clinical modalities, the date of diagnosis will be used as the date of disease progression.
- Overall Survival (OS): Overall survival refers to the date from enrollment to death due to any cause. For patients who are alive at last follow-up, their OS will be censored based on the time of last follow-up. For patients who are lost to follow-up, their OS will be censored based on the last confirmed survival time before loss to follow-up. The OS of data censor is defined as the time from enrollment to censoring.
- R0 resection rate: R0 resection rate refers to the proportion of patients who complete R0 resection in the total number of enrolled patients with surgically resectable gastric cancer through 3 cycles (21 days per cycle) of treatment with Serplulimab in combination with SOX regimen and 9 weeks of thymalfasin. Of these, R0 resection refers to negative microscopic margins.
- Safety of neoadjuvant immunization + chemotherapy + thymalfasin: Observe any adverse events occurred in all patients during the clinical study, including abnormalities in laboratory tests of abnormal clinical symptoms and vital signs, record their clinical manifestations, severity, occurrence time, duration, treatment methods and prognosis, and determine their correlation with the test drug. Drug safety will be evaluated by NCI-CTCAE version 5.0 criteria. Meanwhile, the incidence of Grade 3–4 adverse events will be recorded from the start of neoadjuvant therapy to 30 days after surgery; the proportion of neoadjuvant therapy-related AEs causing surgical delay more than 30 days or inoperable beyond the original plan;
  - 1. **Exploratory endpoints**

Biopsy tissue specimens before neoadjuvant therapy and paraffin-embedded pathological tissue specimens after surgery will be collected from cases meeting the inclusion and exclusion criteria, and the expressions of CD68, CD86, CD163, CD4+T cells, and CD8+T cells will be detected.

1. **Study Design**

This is a prospective, open-label, single-arm phase II clinical study to assess the efficacy and safety of Serplulimab in combination with thymalfasin and SOX regimen for neoadjuvant therapy of locally advanced gastric cancer. It is planned to enroll 30 patients with HER2-negative gastric or gastroesophageal junction adenocarcinoma confirmed by histopathology and/or cytology who were clinically staged as stage III resectable G/GEJ carcinoma (Siewert type III and Siewert type II not requiring combined thoracotomy) as assessed by endoscopic ultrasonography, CT/MRI and other imaging. Endoscopically, gastric tumor tissues will be collected from patients for immunofluorescence detection while clamping gastric tumors for pathological examination.

Preoperatively, 3 cycles of Serplulimab in combination with SOX regimen and 9 weeks of thymalfasin neoadjuvant therapy were administered. Patients underwent imaging examinations after the second and third cycles of neoadjuvant therapy to assess efficacy and the possibility of undergoing radical surgery, and underwent surgery within weeks 2 to 6 from the completion of the third dose. Samples of surgically excised gastric tumor tissues were collected for immunofluorescence. Assessment of postoperative pathological findings included pCR, TRG, MPR, ORR, DCR, clinical downstaging rate and R0 resection rate. Patients' adverse reactions were assessed throughout the neoadjuvant therapy period to test the safety of neoadjuvant immunization combined with chemotherapy. Patients were followed up after surgery and DFS and OS were calculated. In addition, the immune cell infiltration of tumor tissues before and after neoadjuvant therapy was explored to investigate its relationship with the effectiveness of immunotherapy + thymalfasin + SOX.

All eligible subjects received neoadjuvant therapy, which was a neoadjuvant regimen of Serplulimab in combination with thymalfasin and SOX.

- Serplulimab: 300 mg, i.v., D1, Q3W;
- Oxaliplatin: 130 mg/m2, i.v., D1, Q3W;
- Tegafur: oral administration: body surface area < 1.25 m^2^, 40 mg each time; body surface area ≥ 1.25 to < 1.5 m^2^, 50 mg each time; body surface area ≥ 1.5 m^2^, 60 mg each time, twice daily for each treatment cycle at D1-D14;
- Thymalfasin: 4.8 mg, subcutaneous injection, twice a week. On Day 1 and Day 4 of each week during Weeks 1 ~ 9;

After the 2nd treatment course of Serplulimab+SOX and after completion of the 3rd dose before surgery, the investigator will perform 2 assessments to assess whether radical gastrectomy is feasible, and if surgical treatment is feasible, it will be performed within 2-6 weeks after the 3rd dose.

**Study Design Diagram**

**
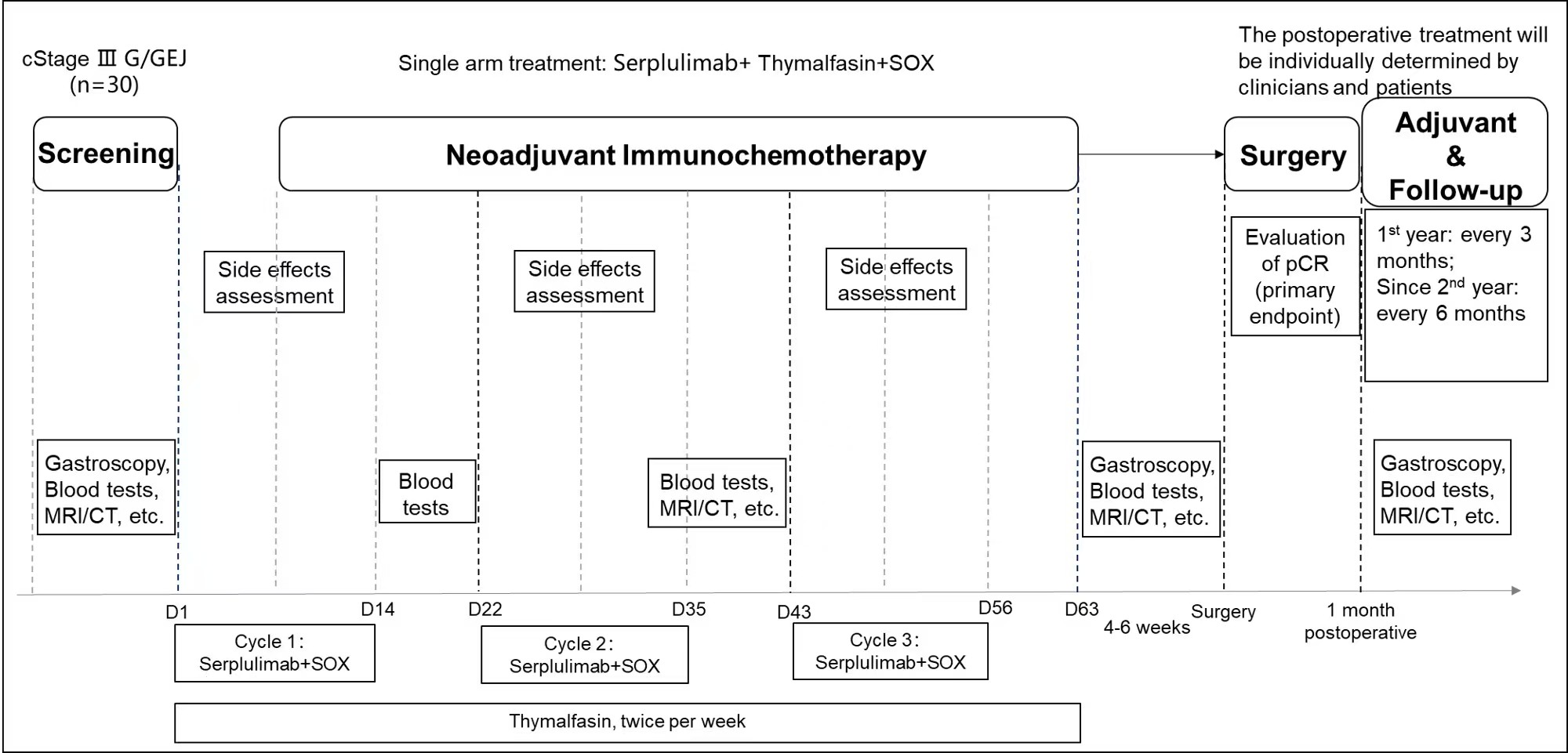
**

1. **Selection Criteria for Subjects**
   1. **Study Population**

cStage III gastric/gastroesophageal junction adenocarcinoma

- 1. **Inclusion Criteria**

1. Male or female patients aged 18–75 years with G/GEJ adenocarcinoma;
2. According to the AJCC 8th edition gastric cancer staging, patients with cStage III (cT_3-4a_N_1-3_M_0_) assessed by abdominal CT and G/GEJ adenocarcinoma diagnosed by gastroscopy and pathology (HER-2 negative or 1+); and patients with gastroesophageal junction (GEJ) cancer will only allow Siewert type III, and Siewert type II subjects who do not require combined thoracotomy to be enrolled.
3. Before enrollment, a doctor in charge of gastrointestinal surgery and an imaging technician jointly assess that the tumor is cStage III and qualified for the study of R0 resection with the purpose of cure, and the patient agrees to receive radical surgery and has no contraindication to surgery as judged by the surgeon;
4. No previous systemic therapy for the current disease, including surgical treatment, anti-tumor chemoradiotherapy/immunotherapy;
5. Expected survival of ≥3 months;
6. There are measurable tumor lesions according to RECIST v1.1 (see Attachment 3 for details);
7. ECOG PS score (see Attachment 4 for details) of 0–1 within 7 days after the first dose;
8. The heart is functioning well and resection for curative purposes can be performed. Patients with underlying ischaemic, valvular or other significant cardiac disease should be evaluated preoperatively by a cardiologist, if clinically indicated;
9. Primary organs are functioning normally and subjects are required to meet the following laboratory parameters:
   1. Absolute neutrophil count (ANC) ≥ 1.5 × 10^9^/L in the absence of granulocyte colony-stimulating factor in the past 14 days;
   2. Platelets ≥ 100 × 10^9^/L without transfusion in the past 14 days;
   3. Hemoglobin > 9 g/dL without or erythropoietin use within the past 14 days;
   4. Total bilirubin ≤ 1.5 × upper limit of normal (ULN); if total bilirubin > 1.5 × ULN but direct bilirubin ≤ ULN, the patient is also allowed to be enrolled;
   5. Aspartate aminotransferase (AST) and alanine aminotransferase (ALT) ≤ 2.5 × ULN
   6. Serum creatinine ≤ 1.5 × ULN and creatinine clearance (calculated using the Cockcroft-Gault formula) ≥ 60 ml/min;
   7. Good coagulation, defined as international normalized ratio (INR) or prothrombin time (PT) ≤ 1.5 times ULN;
   8. Normal thyroid function, defined as thyroid stimulating hormone (TSH) within the normal range. If baseline TSH is outside the normal range, subjects with total T3 (or FT3) and FT4 within the normal range can also be enrolled;
   9. Myocardial enzymes within the normal range (subjects with isolated laboratory abnormalities judged as not clinically significant by the investigator are also allowed to be enrolled);
10. Thyroid function indicators: thyroid stimulating hormone (TSH) and free thyroxine (FT3/FT4) within the normal range or with mild and clinically insignificant abnormalities;
11. Body weight above 40 kg (including 40 kg), or BMI > 18.5;
12. Female patients must meet:

- Patients with menopausal (defined as no menses for at least 1 year and no other confirmed cause other than menopause) status, or who have been surgically sterilized (removal of ovaries and/or uterus), or who are of childbearing potential must also meet the following requirements:
- Pregnancy test must be negative within 7 days prior to first dose;
- Agree to use contraception with an annual failure rate of < 1% or remain abstinent (avoid heterosexual intercourse) from signing of informed consent through at least 120 days after the last dose of study drug and for at least 9 months after surgery (contraceptive methods with an annual failure rate of < 1% include bilateral tubal ligation, male sterilization, correct use of hormonal contraceptives that can inhibit ovulation, hormone-releasing intrauterine devices, and copper-containing intrauterine devices.) ;
- Do not breastfeed.

1. Male patients must meet:

Agree to practice abstinence (avoid heterosexual intercourse) or use contraception as defined below: Male patients must remain abstinent or use condoms correctly for at least 120 days after the last dose of study drug and for at least 9 months after surgery if the partner is a woman of childbearing potential or if the partner is pregnant. Reliability of sexual abstinence should be evaluated with reference to the duration of the clinical study, patient preferences, and lifestyle of daily living. Periodic abstinence (eg, calendar day, ovulation, basal body temperature, or post-ovulation contraceptive methods) and withdrawal are not acceptable methods of contraception;

1. Subjects read and fully understood the patient information and signed the informed consent form.
   1. **Exclusion Criteria**
2. Patients with prior (within 5 years) or concurrent other malignancy. Patients with cured localized tumors, such as cutaneous basal cell carcinoma, cutaneous squamous cell carcinoma, superficial bladder cancer, prostate carcinoma in situ, cervical carcinoma in situ, breast carcinoma in situ, stage I lung cancer, and stage I colorectal cancer, can be enrolled;
3. Patients who are scheduled to undergo or have previously undergone organ or bone marrow transplant;
4. Patients who have received blood transfusion within 2 weeks before the first dose or have a history of bleeding, and experience any bleeding event with a severity of grade 3 or higher based on CTCAE 4.0 within 4 weeks before screening;
5. Patients with abnormal coagulation function and bleeding tendency (INR > 1.5 in the absence of anticoagulants); patients treated with anticoagulants or vitamin K antagonists such as warfarin, heparin or their analogues; patients treated with low-dose warfarin (1 mg orally once daily) or low-dose aspirin (no more than 100 mg daily) for preventive purposes are allowed under the premise of prothrombin time international normalized ratio (INR) ≤ 1.5;
6. Have a history of arterial/venous thrombotic events within 6 months before screening, such as cerebrovascular accident (including transient ischemic attack), deep venous thrombosis (venous thrombosis caused by venous catheterization for previous chemotherapy is excluded if the investigator judges that the patient has recovered) and pulmonary embolism;
7. Have a history of myocardial infarction and poorly controlled arrhythmia within 6 months before the first dose (including QTc interval ≥ 450 ms for males and ≥ 470 ms for females) (QTc interval are calculated with Fridericia formula);
8. Presence of NYHA criteria class III-IV cardiac dysfunction or echocardiography: LVEF (left ventricular ejection fraction) < 50%;
9. Urine routine suggests urine protein ≥ + + and confirms 24-hour urine protein > 1.0 g;
10. There are multiple factors affecting oral medication (such as inability to swallow, chronic diarrhea and intestinal obstruction, etc.);
11. Pleural or peritoneal effusion with clinical symptoms requiring clinical intervention;
12. Human immunodeficiency virus (HIV) infection;
13. Active pulmonary tuberculosis;
14. Chronic unhealed wounds or incompletely healed fractures;
15. Patients with previous and current interstitial pneumonia, pneumoconiosis, radiation pneumonitis, drug-related pneumonia, severely impaired pulmonary function, etc. that may interfere with the detection and treatment of suspected drug-related pulmonary toxicity;
16. Presence of known active or suspected autoimmune disease, except for those who are in a stable state at enrollment (not requiring systemic immunosuppressive therapy);
17. History of severe chronic autoimmune diseases, such as systemic lupus erythematosus; history of inflammatory bowel diseases such as ulcerative enteritis and Crohn 's disease; history of chronic diarrheal diseases such as irritable bowel syndrome; history of sarcoidosis or tuberculosis; history of active hepatitis B and C and HIV-infected patients; patients with well-controlled non-serious immune diseases, such as dermatitis, arthritis, psoriasis, etc., can be enrolled. Patients with hepatitis B virus titer < 500 copies/ml can be enrolled;
18. Patients requiring treatment with systemic corticosteroids (at dose level > 10 mg/day prednisone efficacy) or other immunosuppressive drugs within 14 days prior to the first dose or during the study. However, enrollment is permitted if: In the absence of active autoimmune disease, patients are permitted to use topical or inhaled steroids, or adrenal hormone replacement therapy at dose level ≤ 10 mg/day prednisone efficacy;
19. Any active infection requiring systemic anti-infective treatment within 14 days before the first dose, with the exception of prophylactic antibiotic therapy (eg, prevention of urinary tract infection or chronic obstructive pulmonary disease);
20. Treatment with live vaccines within 28 days prior to the first dose; except for inactivated viral vaccines for seasonal influenza;
21. Prior treatment with antibody/drug therapy against immune checkpoints, such as PD-1, PD-L1, CTLA-4 inhibitors;
22. Treatment with related drugs or medical technology affecting immunity within 6 months prior to the first dose (including but not limited to: thymopentin, thymalfasin, interferon, CAR-T therapy, etc.);
23. Patients who are receiving other clinical study treatment, or planning to start this study treatment less than 1 month before the end of previous clinical study treatment;
24. Known history of allergy or intolerance to any study medications or their components;
25. Patients with a history of alcohol abuse, drug abuse and drug abuse. Patients who have stopped drinking alcohol can be enrolled;
26. Patients who do not follow the doctor 's advice, do not take medicine according to the regulations, or have incomplete data that may affect the efficacy judgment or safety judgment;
27. Pregnant or lactating female patients;
28. Patients with conditions that may increase the risk of study participation and study medications, or other severe, acute, or chronic diseases that, in the judgment of the investigator, will make them inappropriate for participation in a clinical study.
29. Patients with other conditions unsuitable for this clinical trial judged by the investigator.
30. **Study Termination and Withdrawal**
    1. **Termination Criteria**

- Intolerable toxicity that does not resolve despite dose adjustment occurs, which requires discontinuation of the study drug treatment;
- During the study period, other anti-tumor treatments, or any concomitant treatment that may significantly impact the safety and efficacy of the study drug, is required;
- An adverse event or serious adverse event occurs, which requires treatment discontinuation as judged by the investigator;
- The subject experiences other new malignancies;
- The subject experiences disease progression during neoadjuvant therapy and is deemed by the investigator to obtain no further benefit from continuing the trial and is ineligible for radical surgical resection (disease progression during neoadjuvant therapy but still eligible for radical surgical resection after evaluation, with at least one completed cycle of neoadjuvant therapy, shall not be considered as study termination);
- Poor subject compliance affects efficacy and safety assessments, with no improvement after the investigator's communication;
- Major protocol violations;
- Other conditions deemed by the investigator as unsuitable for continued treatment.
  1. **Withdrawal Criteria**
- The subject or his/her legal representative withdraws the informed consent;
- Subject's loss to follow-up;
- Subject's pregnancy;
- Subject's death;
- The investigator deems it medically necessary for the subject to withdraw from the trial.

1. **Study Process**
   1. **Screening Period (≤28 days)**

Within these 28 days, patients must complete a non-contrast chest CT, contrast-enhanced abdominal CT, non-contrast + contrast-enhanced gastric tumor MRI, and endoscopy. Based on imaging and pathological findings, the preoperative clinical staging should be assessed as potentially resectable stage III (cStage III) G/GEJ cancer (for GEJ cancer, endoscopic evaluation must confirm Siewert type III or Siewert type II not requiring combined thoracotomy). In addition, patients must complete examinations required by other inclusion criteria, including echocardiography, 12-lead electrocardiogram (ECG), complete physical examination, measurement of height, weight, and vital signs, calculation of BMI, meet the inclusion criteria, and sign the informed consent form to be formally enrolled.

**After enrollment**:

1. Endoscopically, tumor tissue will be collected from patients for subsequent immunofluorescence assay.
2. Patients should complete hematology, blood biochemistry, urinalysis, coagulation function, tumor markers, thyroid function, cardiac function markers, pre-transfusion eight-item panel, and other blood tests within 7 days (≤7 days) before the first neoadjuvant therapy, and complete ECOG scoring (0–1 points). After evaluation, patients should have no significant contraindications to immunotherapy or chemotherapy, and are eligible for neoadjuvant immunotherapy combined with thymalfasin and chemotherapy (regardless of PD-L1 expression, MMR, or MSI status).
   1. **Neoadjuvant Therapy Period**
3. Adverse reactions after treatment should be assessed within 14 days from the end of each cycle of neoadjuvant therapy to the start of the next cycle, including symptom-directed physical examination, 12-lead electrocardiogram, and ECOG score.
4. Blood tests including hematology, blood biochemistry, urinalysis, coagulation function, tumor markers, thyroid function, and cardiac markers should be completed within 3 days (≤3 days) prior to the second neoadjuvant therapy.
5. A non-contrast chest CT scan and an abdominal contrast-enhanced CT should be performed to evaluate ORR and DCR and adverse reactions of patients should also be assessed within 7 days (≤7 days) before the start of Cycle 3 of neoadjuvant therapy.
6. Blood tests including hematology, blood biochemistry, urinalysis, coagulation function, tumor markers, thyroid function, and cardiac markers should be completed within 3 days (≤3 days) prior to the third neoadjuvant therapy;
7. Abdominal contrast-enhanced CT and non-contrast + contrast-enhanced gastric tumor MRI should be performed within 7 days after completion of neoadjuvant therapy (after Cycle 3) to assess the feasibility of surgery, ORR, and DCR. Blood tests including hematology, blood biochemistry, urinalysis, coagulation function, tumor markers, thyroid function, and cardiac markers, as well as electrocardiogram (ECG) and ECOG scoring should be completed within 7 days (≤7 days) before surgery.
8. The surgery should be performed within Weeks 2–6 after the completion of the last chemotherapy + immunotherapy. The R0 resection rate of surgery in the overall patient population should be assessed. Tumor tissue from surgically resected specimens should be collected for immunofluorescence assay. Postoperative pathological specimens should be evaluated for pCR, MPR, and TRG (marking the conclusion of the study-related treatment). Blood tests such as hematology, blood biochemistry, urinalysis, and coagulation function should be completed within 3–7 days after surgery.

**Neoadjuvant therapy regimen**

- 3 cycles of Serplulimab in combination with SOX regimen (oxaliplatin + Tegafur) and 9 weeks of thymalfasin
- Serplulimab in combination with the SOX regimen (oxaliplatin + Tegafur): every 3 weeks (21 days) as one treatment cycle, for a total of 3 cycles

**The usage and dosage of each study drugs are as follows:**

- Serplulimab: 300 mg/dose, intravenous infusion, D1, once every 3 weeks.
- Thymalfasin: 4.8 mg, subcutaneous injection, twice a week. On Day 1 and Day 4 of each week during Weeks 1–9
- Chemotherapy: SOX regimen

Oxaliplatin: 130 mg/m^2^, intravenous infusion over 2–6 hours (administered after completion of Serplulimab infusion), D1, once every 3 weeks; if the body surface area exceeds 2.0 m^2^, the investigator may calculate the dose based on 2.0 m^2^ according to actual conditions;

Tegafur: Taken orally twice daily from Day 1 to Day 14 (D1–D14) based on body surface area, with 3 weeks (21 days) as one treatment cycle.

**Body surface area (m^2^), first dose (as Tegafur):**

<1.25 m^2^, 40 mg/dose

≥1.25 m^2^ to <1.5 m^2^, 50 mg/dose

≥1.5 m^2^, 60 mg/dose

- 1. **Postoperative Follow-up Period**

1. The occurrence of postoperative complications should be followed up within 30 days after surgery;
2. All enrolled subjects will be continuously monitored: patients who receive surgery will be followed up every 3 months within the first year after surgery and every 6 months thereafter until 2 years, with the following recorded: physical examination, laboratory test results (including tumor marker tests), imaging findings (presence of recurrence or metastasis), adjuvant treatment regimens, and treatment duration.
3. **Use of Study Drugs**
   1. **Serplulimab**

Serplulimab from Shanghai Henlius Biotech, Inc. is used in this study, with a strength of 100 mg/vial. The main active ingredient of Serplulimab is a recombinant fully human anti-programmed death receptor-1 (PD-1) monoclonal antibody, with a concentration of 10 mg/mL. This product is a clear, colorless liquid, free from foreign matter, flocculent material or precipitate. The excipients include citric acid, sodium citrate, sodium chloride, mannitol, polysorbate 80, and water for injection.

The finished product of Serplulimab should be stored protected from light at 2–8℃, with a shelf life of 36 months. If turbidity, precipitation, or other quality issues are observed in the injection solution, the product should be immediately stored in sealed containers and Henlius should be notified immediately.

The intravenous infusion time of Serplulimab should be 30–60 minutes. Do not administer by intravenous bolus or a single rapid intravenous injection. Dilution instructions for the drug prior to administration are as follows:

- Prepare the solution and perform infusion
- Do not shake the vial.
- Allow the vial to return to room temperature (25°C or below) before use.
- After taking it out of the refrigerator, the vial may be stored at room temperature (25°C or below) for up to 24 hours prior to dilution.
- Visually inspect the solution for injection for particulate matter and discoloration prior to administration. This product is a clear to slightly opalescent, colorless to pale yellow liquid, free from foreign matter. If visible particles are observed, the vial should be discarded.
- Transfer 2 vials of this injection (300 mg) into an intravenous infusion bag containing 9 mg/mL (0.9%) sodium chloride solution to achieve a final concentration ranging from 1.5 to 5.0 mg/mL. Gently invert the dilution to mix.
- From the perspective of microbiology, the product must be used immediately after dilution and must not be frozen. Stability studies of this product indicate that it can be stored for 24 hours when protected from light at 2–8℃. This 24-hour period includes a maximum of 6 hours at room temperature (20–25℃) under indoor lighting (the 6-hour period includes administration time). After refrigeration, the vials and/or IV bags must be allowed to return to room temperature before use.
- The infusion set used must be equipped with a sterile, pyrogen-free, low protein-binding in-line filter (pore size 0.2 μm). The infusion time should be within 30 to 60 minutes.
- Do not administer simultaneously with other medications using the same infusion line.

This product is for single use only. Any unused medication remaining in the vials must be discarded.

- 1. **Thymalfasin**

Thymalfasin (brand name: Zadaxin) from SciClone Pharmaceuticals (China) Co., Ltd., with a strength of 1.6 mg, is used in this study. The storage, preparation, and administration will be performed in accordance with the instructions provided in the product package insert.

- 1. **Oxaliplatin and Tegafur**

Storage, preparation, and administration should be performed in accordance with the approved product package insert for oxaliplatin and Tegafur.

1. **Dose Adjustments**
   1. **General Principles**

If an adverse event occurs during the study, the investigator will first determine the potential causative drug and adjust the medication based on the most severe adverse event observed in the previous dosing cycle. Prior to administration of the study drug on Day 1 of each cycle, the results of hematology and hepatic and renal function of subjects must meet the requirements for dosing, and all other toxicities must have resolved to CTCAE Grade 0–1 or baseline levels (except for alopecia, fatigue, protocol-specified exceptions, or other conditions deemed clinically insignificant by the investigator). If subjects fail to meet the medication criteria within the planned dosing interval due to adverse events, the next administration time may be delayed. If the investigator confirms that the adverse event is solely caused by one drug, the dose of that specific drug may be adjusted exclusively. If it is unclear whether the adverse reaction is related to two or more drugs, the doses of all potentially relevant drugs may be adjusted simultaneously. If a study drug needs to be temporarily suspended or permanently discontinued due to drug-related toxicity, drug-related adverse events (AEs), or other reasons, other study drugs may be administered alone provided the corresponding medication criteria are met.

All dose adjustment interventions must be documented, including the reasons and actions taken.

- 1. **Doe Adjustment of Serplulimab**

Throughout the study, dose adjustments of Serplulimab are not permitted. The principles for temporary suspension and permanent discontinuation of Serplulimab are outlined in the table below.

Table. Dose Adjustment Scheme for Serplulimab in Case of Adverse Events

| **Immune-related adverse reactions** | **Severity** | **Treatment adjustment regimen** |
| --- | --- | --- |
| Pneumonia | Grade 2 | Treatment suspension until adverse reactions resolve to Grade 0–1. |
|  | Grade 3 or 4 or recurrent Grade 2 | Permanent discontinuation |
| Diarrhea and colitis | Grade 2 or 3 | Treatment suspension until adverse reactions resolve to Grade 0–1. |
|  | Grade 4 | Permanent discontinuation |
| Hepatitis | Grade 2, aspartate aminotransferase (AST) and alanine aminotransferase (ALT) at 3–5 times the upper limit of normal (ULN) or total bilirubin (TBL) at 1.5–3 times the ULN | Treatment suspension until adverse reactions resolve to Grade 0–1. |
|  | Grade 3 or 4, AST or ALT >5×ULN, or total bilirubin >3×ULN | Permanent discontinuation |
| Nephritis | Grade 2 or 3 blood creatinine increased | Treatment suspension until adverse reactions resolve to Grade 0–1. |
|  | Grade 4 blood creatinine increased | Permanent discontinuation |
| Endocrine disorders | Symptomatic Grade 2 or 3 hypothyroidism, Grade 2 or 3 hyperthyroidism, Grade 2 or 3 hypophysitis, Grade 2 adrenal insufficiency, Grade 3 hyperglycemia, or type 1 diabetes mellitus | Treatment suspension until adverse reactions resolve to Grade 0–1. |
|  | Grade 4 hypothyroidism  Grade 4 hyperthyroidism  Grade 4 hypophysitis  Grade 3 or 4 adrenal insufficiency  Grade 4 hyperglycemia | Permanent discontinuation |
| Skin adverse reaction | Grade 3 | Treatment suspension until adverse reactions resolve to Grade 0–1. |
|  | Grade 4, Stevens-Johnson syndrome (SJS) or toxic epidermal necrolysis (TEN) | Permanent discontinuation |
| Platelet count decreased or white blood cell count decreased | Grade 3 | Treatment suspension until adverse reactions resolve to Grade 0–1. |
|  | Grade 4 | Permanent discontinuation |
| Other immune-related adverse reactions | Grade 3 or 4 blood amylase increased or lipase increased  Grade 2 or 3 pancreatitis  Grade 2 myocarditis*  Other Grade 2 or 3 immune-related adverse reactions occurring for the first time | Treatment suspension until adverse reactions resolve to Grade 0–1. |
|  | Grade 4 pancreatitis or pancreatitis recurrent of any grade  Grade 3 or 4 myocarditis  Grade 3 or 4 encephalitis  Other Grade 4 immune-related adverse reactions occurring for the first time | Permanent discontinuation |
| Recurrent or persistent adverse reactions | Recurrent Grade 3 or 4 (excluding endocrine disorders)  Grade 2 or 3 adverse reactions not improved to Grade 0–1 within 12 weeks after the last dose (excluding endocrine disorders)  Corticosteroids not reduced to ≤10 mg/day prednisone equivalent dose within 12 weeks after the last dose | Permanent discontinuation |
| Infusion reaction | Grade 2 | Infusion rate reduction or dose suspension; medication may be resumed when symptoms alleviate, along with close monitoring |
|  | Grade 3 or 4 | Permanent discontinuation |

Note: The severity of adverse reactions is assessed according to the National Cancer Institute Common Terminology Criteria for Adverse Events Version 5.0 (NCI-CTCAE v5.0).

*The safety of resuming this product after myocarditis has improved to Grade 0–1 with treatment is unknown.

- 1. **Management of Infusion-Related Reactions Associated with Serplulimab**

Serplulimab may cause severe or life-threatening infusion reactions, including severe hypersensitivity or anaphylaxis. Signs and symptoms typically occur during or shortly after drug infusion and usually resolve completely within 24 hours after the infusion is completed. Guidelines for the management of infusion-related reactions associated with Serplulimab are detailed in the table below.

Table. Guidelines for the Management of Infusion-Related Reactions Associated with Serplulimab

| NCI CTCAE Grade | Treatment | Premedication for subsequent dosing |
| --- | --- | --- |
| Grade 1  Mild reactions; no infusion interruption not indicated; intervention not indicated | Based on the patient's medical indications, monitoring of vital signs should be intensified until the investigator deems the subject's condition to be stable. | None |
| Grade 2  Therapy or infusion interruption indicated but responds promptly to symptomatic treatment (for example, antihistamines, NSAIDs, narcotics, IV fluids); prophylactic medications indicated for ≤24 h. | The infusion should be discontinued and symptoms should be monitored.  Other appropriate drug treatments may include, but are not limited to:  Intravenous infusion  Antihistamines  NSAIDS  Acetaminophen anesthetics  Based on the patient's medical indications, monitoring of vital signs should be intensified until the investigator deems the subject's condition to be stable.  If symptoms resolve within one hour after discontinuation of the drug infusion, the infusion may be restarted at 50% of the original rate (e.g., reduced from 100 mL/h to 50 mL/h). Otherwise, administration should be suspended until symptoms resolve, and the subject should receive premedication prior to the next scheduled dose.  For subjects who experience Grade 2 toxicity despite adequate premedication, further treatment with study drugs should be permanently discontinued. | Subjects may receive the following premedications at 1.5 hours (±30 minutes) prior to the infusion of Serplulimab:  50 mg diphenhydramine taken orally (or an equivalent dose of antihistamines).  500–1000 mg of acetaminophen taken orally (or an equivalent dose of antipyretics). |
| Grade 3 or 4  Grade 3:  Prolonged duration (i.e., not rapidly responsive to symptomatic treatment and/or short interruption of infusion); recurrence of symptoms following initial improvement; hospitalization indicated for other clinical sequelae (e.g., renal impairment, pulmonary infiltration)  Grade 3:  Life-threatening; requiring vasopressor therapy or ventilatory support | Infusion discontinuation.  Other appropriate drug treatments may include, but are not limited to:  Epinephrine**  Intravenous infusion  Antihistamines  NSAIDS  Acetaminophen anesthetics  Oxygen  Vasopressors  Corticosteroids  Based on the patient's medical indications, monitoring of vital signs should be intensified until the investigator deems the subject's condition to be stable.  Hospitalization may be required.  **In case of an allergic reaction, epinephrine should be administered immediately.  Subjects should permanently discontinue further treatment with study drugs. | No further dosing |
| Appropriate emergency equipment should be available in the ward, and physicians must be readily accessible during drug administration. | | |

- 1. **Other Approved Dose Adjustments of Serplulimab**

In addition to treatment-related AEs, Serplulimab treatment may be suspended due to conditions not related to study treatment, such as medical/surgical events or administrative reasons. Subjects should resume study treatment within 3 weeks after the planned treatment suspension or as determined by the investigator. The reasons for treatment suspension should be documented in the study records of the patients.

- 1. **Dose Adjustment of Tegafur and Oxaliplatin**

In case of intolerance to the SOX regimen, dose adjustments are permitted (dose adjustments are to be implemented according to the investigator based on clinical conditions or with reference to Tables 1 through 4). Regarding Tegafur, the dose may be appropriately increased or decreased based on the patients' conditions, with dose levels set at 40, 50, 60, and 75 mg/dose. When the dose needs to be increased, if no abnormalities in clinical test values (hematology, liver and kidney function tests) or gastrointestinal symptoms related to this product are observed, and there are no safety concerns, the dose may be sequentially increased by one level from the baseline dose, with a maximum single dose limit of 75 mg/dose. When the dose needs to be decreased, it should be sequentially reduced by one level from the baseline dose, with a minimum single dose limit of 40 mg/dose.

**Table 1. Adjustment Principles for Chemotherapy Drugs**

| **Dose level** | **Oxaliplatin administration regimen** | **Tegafur dose regimen** |
| --- | --- | --- |
| Starting dose | 130 mg/m^2^  Intravenous infusion  Administered on Day 1 of each cycle.  Administered once every 3 weeks (21 days) | Based on the body surface area (calculated as Tegafur)  <1.25 m^2^, 40 mg/dose;  ≥1.25 m^2^ to <l.5 m^2^, 50 mg/dose;  ≥1.5 m^2^, 60 mg/dose orally  Twice daily, D1–D14 |
| First dose reduction | 75% of the starting dose | Initial dose of 60 mg: dose reduced to 50 mg;  Initial dose of 50 mg: dose reduced to 40 mg;  Initial dose of 40 mg: dose discontinue |
| Second dose reduction | - | Initial dose of 60 mg: dose reduced to 40 mg;  Initial dose of 50 mg: dose discontinue |
| Third dose reduction | - | Initial dose of 60 mg: dose discontinue |

**Table 2. Dose Adjustments for Hematologic Toxicity**

| **Type (NCI-CTC)** | **Starting dose** | |
| --- | --- | --- |
|  | **Tegafur** | **Oxaliplatin** |
|  | **Dose adjustments of study drugs** | |
| **Neutropenia** |  |  |
| Grade 3 or 4 | The dose should be sequentially reduced to 50 mg/dose, 40 mg/dose, or discontinuation based on the initial dose | 75% of the original dose |
| **Thrombocytopenia** |  |  |
| Grade 3 or 4 | The dose should be sequentially reduced to 50 mg/dose, 40 mg/dose, or discontinuation based on the initial dose | 75% of the original dose |
| **Febrile neutropenia** |  |  |
| Grade 3 | The dose should be sequentially reduced to 50 mg/dose, 40 mg/dose, or discontinuation based on the initial dose | 75% of the original dose |
| Grade 4 | Discontinuation | Discontinuation |

**Table 3. Dose Adjustments for Non-Hematologic Toxicity**

| **Type (NCI-CTC)** | Starting dose | |
| --- | --- | --- |
|  | **Tegafur** | **Oxaliplatin** |
|  | Dose adjustments of study drugs | |
| Grade 4 nausea and/or vomiting |  | Treatment discontinuation |
| Diarrhea |  |  |
| Grade 3 | The dose should be sequentially reduced to 50 mg/dose, 40 mg/dose, or discontinuation based on the initial dose | None |
| Grade 4 | The dose should be sequentially reduced to 50 mg/dose, 40 mg/dose, or discontinuation based on the initial dose | 75% of the original dose |
| Stomatitis |  |  |
| Grade 3 | The dose should be sequentially reduced to 50 mg/dose, 40 mg/dose, or discontinuation based on the initial dose | None |
| Grade 4 | The dose should be sequentially reduced to 50 mg/dose, 40 mg/dose, or discontinuation based on the initial dose | 75% of the original dose |
| ≥ Grade 2 cardiotoxicity | Treatment discontinuation | |
| Grade 3 or 4 hand and foot skin reactions | The dose should be sequentially reduced to 50 mg/dose, 40 mg/dose, or discontinuation based on the initial dose | None |
| Grade 3 or 4 allergic reactions | Treatment discontinuation | |
| Cerebellar neurotoxicity | Treatment discontinuation | |
| Other clearly drug-related toxicities |  | |
| Grade 3 | The dose should be sequentially reduced to 50 mg/dose, 40 mg/dose, or discontinuation based on the initial dose | 75% of the original dose |
| Grade 4 | Treatment discontinuation | |

**Table 4. Dose Adjustments for Oxaliplatin-Induced Neurotoxicity**

| **Toxicity** | **Grade** | **Duration of Toxicity** | | |
| --- | --- | --- | --- | --- |
|  |  | **1–7 days** | **>7 days** | **Persisting between cycles ^a^** |
| Paresthesia, dysesthesia^b^, not affecting function | 1 | No dose adjustment required | No dose adjustment required | No dose adjustment required |
| Paresthesia, dysesthesia^b^, affecting function but not interfering with activities of daily living (ADL) | 2 | No dose adjustment required | No dose adjustment required | 100mg/m^2^ |
| Paresthesia, dysesthesia^b^, accompanied by pain or functional impairment, affecting ADL. | 3 | No dose adjustment required | 100mg/m^2^ | Permanent discontinuation of treatment |
| Persistent paresthesia, dysesthesia, disabling or life-threatening | 4 | Permanent discontinuation of treatment | Permanent discontinuation of treatment | Permanent discontinuation of treatment |
| Acute: (during infusion or within 2 hours) abnormal sensation in the throat ^b^ |  | Extend the next drug infusion time to 6 h | N/A | N/A |
| - 1. Not resolved at the start of the next cycle.   2. May be cold-induced. | | | | |

- 1. **Principles for Managing Toxicity of Immune Checkpoint Inhibitor**

Adverse events (AEs) associated with exposure to Serplulimab may have an immunological etiology, attributable to its mechanism of action in blocking the interaction between PD-1 and PD-L1, thereby restoring T-cell activity. This may lead to hyperactive immune function, resulting in immune-related adverse events (irAEs). These irAEs may occur shortly after the first dose or months after the last dose of Serplulimab and simultaneously affect more than one body system, such as immune-related pneumonitis, diarrhea/colitis, renal insufficiency, rash, hepatitis, endocrine disorders, and autoimmune AEs like peripheral or central neuritis. Therefore, early identification and initiation of treatment are crucial for reducing complications. Based on available clinical trial data, most irAEs are reversible and can be managed by interrupting Serplulimab administration, administering glucocorticoids, and/or providing other supportive therapies. Subjects who experience the aforementioned AEs in this study should be monitored for symptoms and signs. Relevant examinations such as bronchoscopy, endoscopy, or skin biopsy should be performed to determine the etiology. If no alternative etiology is identified (such as disease progression, concomitant medications, and infections) and treatment with corticosteroids and/or other immunosuppressants is required (excluding endocrine events such as hyperthyroidism/hypothyroidism, hypophysitis, type 1 diabetes mellitus, and adrenal insufficiency, which may not require immunosuppressive therapy but are still considered to be related to the hyperactive immune function induced by Serplulimab), the aforementioned AEs should be deemed associated with hyperactive immune function caused by Serplulimab and diagnosed as irAEs. Based on the severity of irAEs, Serplulimab treatment may be temporarily suspended or permanently discontinued, and corticosteroids may be administered.

- 1. **Concomitant Medications and Concomitant Therapies**
     1. **Permitted Concomitant Medications/Concomitant Therapies**
  - Medications that are judged by the investigator to be consistent with the protocol (e.g., for treatment of disease-related symptoms and concomitant therapy for treatment-related AEs).
  - Subjects who require long-term medication due to underlying diseases such as hypertension and diabetes mellitus may continue to take the drug.
  - Topical glucocorticoid administration, such as topical skin application, eye drops, nasal spray, inhalation, is allowed.
    1. **Contraindicated Concomitant Medications/Therapies**
  - During the treatment period of this study, subjects are prohibited from receiving the following treatments:
  - Biological therapies with anti-tumor effects (excluding cytokine drugs used to treat adverse events caused by chemotherapeutic agents), as well as traditional Chinese medicinal products with anti-tumor effects.
  - Immunomodulatory drugs not specified in this protocol, including but not limited to non-specific immunomodulators (such as thymosin, interferon, interleukin, immunoglobulin, gamma globulin) and traditional Chinese medicinal products with immunomodulatory effects.
  - Chemotherapy or targeted therapy not specified in this protocol
  - Live vaccinations within 30 days prior to the first dose of Serplulimab and during the study. Live vaccines, including but not limited to: vaccines against measles, mumps, rubella, varicella, yellow fever, rabies, BCG, and typhoid fever (oral). Vaccination with inactivated virus vaccines against seasonal influenza via injection is permitted, but live attenuated influenza vaccine for nasal spray is not allowed.
  - Corticosteroids. Inhaled corticosteroids as part of fixed treatment for asthma or chronic obstructive pulmonary disease (COPD) are permitted; corticosteroids for managing immune-related adverse events are permitted; physiological doses of corticosteroids may be approved after consultation with the sponsor.
  - Note: Prophylactic corticosteroids may be used to prevent allergic reactions (e.g., pretreatment before administration of intravenous contrast agents or chemotherapeutic drugs).
  - Subjects who, in the investigator's assessment, require any of the aforementioned treatment methods for clinical management should be excluded from the study. Subjects may receive other drug treatments deemed medically necessary by the investigator.
  - It is crucial for the investigator to review all drugs (both prescription drugs and over-the-counter drugs) that subjects receive before the study begins and at each study visit.
  - At each visit, any new drug received by subjects must be inquired about.
  - To minimize the risk of adverse drug interactions, all necessary measures must be taken to limit the number of concomitant medications that are truly necessary.
  - During the treatment period, drugs with hepatotoxicity (i.e., as explicitly listed in the drug package insert with warnings for hepatotoxicity) should be avoided.

1. **Management of Study Drugs**
   1. **Storage and Management of Study Drugs**

Serplulimab should be stored refrigerated at 2–8℃, protected from light and moisture; freezing is strictly prohibited. Zadaxin should be protected from light and stored at 2–8℃. All study drugs, including Serplulimab and Zadaxin, should be transported to each study site via cold chain. The study site must designate a dedicated person responsible for the storage and dispensing of the study drugs.

The study drugs Serplulimab and Zadaxin should be stored in a refrigerator accessible only to authorized personnel. Upon receipt of the drugs, the investigator must verify that the transportation temperature is within the specified range. After confirmation, the investigator should sign for the drugs and store them at the specified temperature. If temperature deviations occur during transportation or storage at the study site, the drugs should be promptly isolated and transferred to the specified temperature conditions. The drugs should not be administered to subjects temporarily, and the incident must be reported immediately for further handling according to the instructions of Henlius and SciClone.

- 1. **Drug Recovery and Destruction**

In this study, containers for used study drug may be destroyed on-site in accordance with the applicable guidelines and standard operating procedures established by the study site and local institutions. All unused study drugs must be collected and uniformly destroyed upon study completion/termination or after the expiry date.

- 1. **Records of Study Drugs**

The designated personnel at the study site must promptly document the receipt, distribution, usage, inventory, destruction, recovery, and damage of study drugs in accordance with relevant regulations and guidelines.

1. **Efficacy Evaluation**

Imaging examinations will be performed to evaluate efficacy and feasibility of surgery after the completion of Cycles 2 and 3 of neoadjuvant therapy (within 7 days before the start of Cycle 3 and within 7 days after the end of Cycle 3). Imaging examinations are conducted after surgery and prior to adjuvant therapy, and pathological assessment will also be performed to evaluate the completeness of surgery. Evaluation requires a full abdominal direct contrast-enhanced (iodinated contrast) CT scan and gastric tumor MRI (contrast-enhanced). The same imaging technique should be used for pre- and post-evaluation in the same patient, and all imaging data must be retained. Patients who experience PD may continue the study treatment if they are willing and deemed suitable by the investigator to continue the original treatment regimen after re-signing the informed consent form, and re-evaluation will be performed after 4 weeks; otherwise, the patients will discontinue the study. For patients who discontinue treatment due to intolerable toxicity, if no efficacy evaluation is performed within 4 weeks prior to discontinuation, an efficacy evaluation should be conducted at the time of discontinuation.

For patients with potentially resectable gastric cancer after neoadjuvant therapy, surgery is scheduled within Weeks 2–6 after the completion of the last chemotherapy + immunotherapy. The surgical approach is determined based on the degree of disease response, including R0 resection, R1 resection, or cytoreductive surgery. The postoperative efficacy evaluation criteria primarily focus on surgical pathological assessment (complete pathological response rate, major pathological response rate, tumor regression grade, R0 resection rate, etc.), supplemented by imaging evaluation (objective response rate, disease control rate) for reference. At this point, the study has achieved its primary endpoint.

1. **Safety Reports and Adverse Event Management**
   1. **Definition of Adverse Event**

An adverse event (AE) is defined as any unfavorable and unintended medical occurrence in a clinical study participant from the time of signing the informed consent form, whether or not considered related to the study drug. AEs include, but are not limited to, the following circumstances:

- Exacerbation of pre-existing (prior to entering the clinical trial) medical conditions/diseases (including exacerbation of symptoms, signs, and abnormal laboratory findings);
- Any new adverse medical condition (including symptoms, signs, newly diagnosed diseases);
- Clinically significant abnormal laboratory findings.
  1. **Definition of Serious Adverse Events**

A serious adverse event (SAE) refers to any AE that meets at least one of the following criteria:

- Resulting in death, excluding deaths due to disease progression of the studied indication.
- Life-threatening (the term "life-threatening" in the definition refers to an AE that places the subject at immediate risk of death when it occurs, and does not include AEs that may lead to death only if they worsen).
- Requiring inpatient hospitalization or prolongation of existing hospitalization, excluding:
- Hospitalization in rehabilitation facility
- Hospitalization in sanatorium
- Routine admission by emergency department
- Day surgery (e.g., outpatient/day/ambulatory surgery)
- Hospitalization or prolonged hospitalization not related to the worsening of an AE is not considered an SAE. For example: Hospitalization due to pre-existing conditions without the occurrence of new AEs or exacerbation of the original disease (e.g., abnormal laboratory results persisting before the clinical trial); hospitalization for other reasons (e.g., annual routine physical examinations); hospitalizations specified by the clinical study protocol during the study (e.g., procedures required by the protocol); elective hospitalizations not related to the worsening of AEs (e.g., elective surgeries); scheduled treatments or surgical procedures (should be documented in the overall trial protocol and/or the subject's baseline data); hospitalization solely for the administration of blood products.
- Resulting in persistent or significant disability/incapacity.
- Lead to congenital anomaly/birth defect.
- Other important medical events: defined as events that jeopardize the subject or require medical intervention to prevent any of the aforementioned situations.
  1. **Definition of Adverse Event**

The investigator will evaluate all AEs according to the National Cancer Institute Common Terminology Criteria for Adverse Events (CTCAE) Version 5.0. Any AEs with CTCAE grade changes will be recorded in the Adverse Event Case Report Form/Worksheet.

All AEs, regardless of CTCAE grade, must be evaluated for whether they constitute SAEs.

Evaluation criteria for AEs are as follows:

Table. Evaluation Criteria for Adverse Events

| V5.0 CTCAE  Grading | Grade 1 | Mild; asymptomatic or mild symptoms; clinical or diagnostic observations only; intervention not indicated |
| --- | --- | --- |
|  | Grade 2 | Moderate; minimal, local or noninvasive intervention indicated; limiting age appropriate instrumental ADL |
|  | Grade 3 | Severe or medically significant but not immediately life-threatening; hospitalization or prolongation of hospitalization indicated; disabling; limiting self-care ADL |
|  | Grade 4 | Life-threatening consequences; urgent intervention indicated |
|  | Grade 5 | Death related to AE |
| Severity | A SAE is any of the following AEs occurring at any dose or during the use of any study drug that: | |
|  | - Results in death | |
|  | - Is life threatening; or in the investigator's opinion, the occurrence of the event places the subject at immediate risk of death (Note: This does not include an AE which hypothetically might have caused death if it were more severe); | |
|  | - Results in persistent or significant disability/incapacity; (substantial disruption of one's ability to conduct normal life functions); | |
|  | - Requires inpatient hospitalization or prolongation of existing hospitalization; (Hospitalization is defined as an inpatient admission, regardless of length of stay, even if the hospitalization is a precautionary measure for continued observation. Note: Hospitalization (including hospitalization for an elective procedure) for a pre-existing condition which has not worsened during the study does not constitute a SAE. Pre-existing diseases refer to clinical conditions diagnosed prior to the administration of study drugs, as documented in the patient's medical history); | |
|  | - Is a congenital anomalies/birth defect (in offspring of subjects taking the product, regardless of time of diagnosis); | |
|  | Other important medical events; although an event does not result in death, is not life-threatening, or does not require hospitalization, it may still be considered as a SAE based on appropriate medical judgment if it jeopardizes the subject and may necessitate medical or surgical intervention to prevent one of the outcomes listed above (marked with †). | |
| Duration | The start and end dates of AEs should be recorded. If less than 1 day, the appropriate time duration and unit should be specified. | |
| Measures taken | Does the AE associated with the study drugs lead to discontinuation of the study drug? | |
| Relationship to the study drug | Does the study drug cause the AE? The investigator with medical qualifications must provide the causality assessment results between the study drug and AEs. The investigator will sign/date (with initials) the original documents or worksheets to support the causality assessment on the AE form, ensuring that a medically qualified causality assessment is conducted. This signed document must be retained within the required regulatory time frame. The purpose of the following criteria is to serve as a reference guide to assist the investigator in evaluating the relationship between the study drug and the AE based on available information.  The following factors are used to evaluate the relationship between the study drug and the AE; the greater the correlation between the items and their corresponding factors (in terms of quantity and/or intensity), the higher the likelihood that the AE is caused by the study drug. | |
|  | Exposure | Is there evidence that the subject has been indeed exposed to the study drug, such as: credible past medical history, acceptable compliance assessment (drug count, logs, etc.), expected pharmacological effects, or measurement of the drug/metabolites in biological specimens collected *in vivo*? |
|  | Time course | Is there a reasonable temporal relationship between the AE and the administration of the study drug?  Is the onset time of the AE consistent with a drug-induced AE? |
|  | Possible causes | Could the adverse event not be explained by other etiologies, such as underlying conditions, other medications/vaccines, or host/environmental factors? |
|  | De-challenge test | Whether the study drug has been discontinued or its dose/exposure/frequency has been reduced?  If yes, can the AE be resolved or improved?  If yes, the de-challenge test result is positive. If no, the de-challenge test result is negative.  Note: This criterion does not apply under the following circumstances: (1) the AE results in death or persistent disability; (2) the AE resolves/improves despite continued use of the study drug; (3) the study is a single-dose study; (4) the study drug is administered only once. |
|  | Re-challenge test | Is the subject repeatedly exposed to the study drug in this study?  If yes, is AE recurrent or worsening?  If yes, the re-challenge test result is positive. If no, the re-challenge test result is negative.  Note: This criterion does not apply under the following circumstances: (1) the initial AE results in death or persistent disability, (2) the study is a single-dose study, or (3) the study drug is administered only once.  Note: If a re-challenge test is planned for a SAE that may be caused by the study drug, or if re-exposure to the study drug may pose a serious potential risk to the subject/patient, re-challenge is not recommended unless continuation of the drug is considered beneficial to the patient and no alternative treatment is available. Such re-challenge may only proceed with prior approval from the principal investigator. |
|  | Consistency with the characteristics of the study treatment | Are the clinical/pathological manifestations of the AEs consistent with previous therapeutic data on the study drug or pharmacological and toxicological tests of similar drugs? |
| A medically qualified investigator will assess the relationship based on his/her best clinical judgment, taking into account the aforementioned factors, and report the evaluation results in the case report form/worksheet. | | |
| Recording of causality | | The following table may be used to assess causality (not all criteria need to be met). |
| Related | | There is evidence of exposure to the study drug. The temporal relationship between the occurrence of AE and the administration of the study drug is reasonable. Compared to other causes, the AE is more likely to be attributed to the study drug rather than other factors. |
| Not related | | The subject does not use the study drug, or the temporal relationship between the administration of the study drug and the occurrence of the AE is unreasonable, or there are other reasons more likely to explain the AE than the study drug (also applicable to subjects who have overdose but do not experience related AEs). |

- 1. **Recording of Adverse Events**

The investigator should use medical terminology/concepts to document AEs or SAEs. The use of colloquialisms and abbreviations should be avoided. All AEs (including SAEs) should be recorded in the Adverse Event Form of the CRF.

- - 1. **Adverse event collection and time frame**

The investigator will ascertain AEs by asking subjects non-leading questions.

All AEs, including SAEs, observed by the investigator or spontaneously reported by subjects from the signing of the informed consent form until 30 days post-surgery, will be collected.

- - 1. **Follow-up of adverse events**

AEs should be followed up until they return to baseline or Grade 0–1, or until the investigator deems further follow-up unnecessary for justified reasons (e.g., the AE cannot resolve or has improved). If the AE cannot resolve, a reasonable explanation must be documented in the CRF. Regardless of whether it is related to the study drug, the recovery status and date of the subject's AE or SAE should be recorded in the CRF and medical records.

- - 1. **Recording content of AEs**

The investigator must fully document any AE, including diagnosis (or symptoms, signs, and abnormal laboratory findings if no diagnosis is available), start and end dates and times (if applicable), CTCAE severity grade and changes (for Grade 3 or higher events), whether it is a SAE, measures taken regarding the study drug, treatments administered for the AE, outcome of the event, and the relationship between the AE and the study drug.

For any SAE, the investigator should also provide the date when the AE meets SAE criteria, the date when the investigator becomes aware of the SAE, the basis for classifying the AE as an SAE, dates of hospitalization and discharge, possible cause of death, date of death, whether an autopsy is performed, causality assessment with the study procedures, causality assessment with other medications, and other potential causes leading to the SAE.

The investigator should also provide the rationale for causality assessment and a description of the SAE. In the SAE description, the following must be included: subject No., age, sex, height, and weight; the indication for the study drug treatment, disease stage, and relevant systemic conditions; the clinical course of the SAE, including its onset, progression, outcome, and final result; laboratory test results related to the SAE (the test time, units, and normal reference ranges must be provided); medical history related to the SAE, comorbidities, and their onset and duration; medication history related to the SAE, concomitant medications, and details of their initiation, duration, and dosage and administration; as well as detailed information on the study drug treatment, including its initiation, duration, dosage and administration.

The description of AE records is as follows:

**Diagnosis, symptoms, and signs**

If a diagnosis has already been established, the diagnosis rather than individual symptoms and signs should be recorded on the CRF (e.g., record hepatic failure instead of jaundice, transaminases increased, and flapping tremor). If the symptoms and signs cannot be definitively attributed to this diagnosis at the time of reporting, they should be recorded as separate AEs/SAEs. If it is determined that the symptoms and signs are caused by the diagnosis, only the diagnosis should be reported alone, with the symptoms and signs being included in the diagnosis. For AEs, records of symptoms and signs should be deleted; for SAEs, follow-up update reports must be submitted.

**Adverse events secondary to other events**

Generally, for AEs secondary to other events (e.g., caused by other events or clinical sequelae), the primary events should be recorded, unless the secondary events are serious or constitute SAEs. However, clinically significant secondary events should be recorded as independent AEs in the CRF if they occur at different times from the primary events. If the relationship between events is unclear, the events should be recorded separately in the eCRF.

**Persistent or recurrent adverse events**

Persistent AEs refer to those that continue to exist without resolution between two evaluation time points for subjects. Such AEs should be recorded only once in the CRF. The initial severity of the event should be recorded and updated when the event worsens to document the highest severity.

Recurrent AEs refer to those that have resolved between two evaluation time points but subsequently recur. The occurrence of such events should be recorded separately in the CRF.

**Abnormal laboratory findings**

Clinically significant abnormal laboratory findings should be reported as AEs. The investigator is responsible for reviewing all abnormal laboratory findings and making a medical judgment on whether each laboratory abnormality should be reported as an AE.

**Death**

All deaths occurring during the entire study, including the 30-day follow-up period after the last dose, regardless of their relationship to the study drug, should be recorded in the Death Report Form of the CRF and promptly reported to the sponsor.

When recording death events, if the cause of death is clearly identified, the cause of death shall be documented as an AE, with the outcome of the AE being death, and the event shall be reported as a SAE (deaths due to tumor progression or deaths occurring 30 days after the last dose that are considered not related to the study drug, and are not recorded or reported as AEs/SAEs; however, the investigator shall document the death in the Death Report Form of the CRF and promptly notify the sponsor). If the cause of death is unknown at the time of reporting, it shall be recorded as "death of unknown cause" in the Adverse Event Form of the CRF, and "death of unknown cause" shall initially be reported as an SAE, followed by further investigation to determine the exact cause of death.

**Pre-existing medical conditions**

Any pre-existing symptoms/signs in subjects during the study screening period should only be recorded and reported as AEs upon entering the study if there is an increase in severity, frequency, or nature (excluding worsening of the studied disease condition). The record should reflect changes relative to the previous condition, such as "increased frequency of headaches."

**Disease progression**

Disease progression is defined as the worsening of the subject's condition due to the primary tumor targeted by the study drug; the emergence of new lesions relative to the primary tumor or the progression of existing lesions is considered disease progression. Expected disease progression is not reported as an AE. Death, life-threatening conditions, hospitalization or prolonged hospitalization, persistent or significant disability/incapacity, congenital anomalies/birth defects, or other important medical events resulting from symptoms and signs of expected disease progression are not subject to expedited reporting as SAEs.

- 1. **Expedited Reporting of SAEs and Pregnancy**

**Reporting of SAEs:**

The reporting period for SAEs is from the signing of the informed consent to 30 days after surgery (inclusive). In the event of an SAE, whether it is an initial report or a follow-up report, the investigator must promptly complete the Serious Adverse Event Report Form, sign and date it, and report it to the sponsor within 24 hours of becoming aware of the event. Meanwhile, SAEs occurring after using Henlius products shall be reported to Henlius: (HenliusPVG@Henlius.com) within 24 hours after awareness, and the SAE occurring after using SciClone products shall be reported to SciClone (PVCHINA@sciclone.com) within 24 hours after awareness.

If a SAE occurring outside the aforementioned period is determined to be related to the study drug, it should also be reported.

**Pregnancy:**

As similar drugs have been associated with embryotoxic safety risks, all subjects of childbearing potential participating in the clinical study must take effective contraceptive measures.

If a female subject becomes pregnant during the clinical study while exposed to the study drug, the subject will be withdrawn from the study. The sponsor, Henlius, and SciClone's pharmacovigilance departments must be notified within 24 hours of the investigator's awareness of the pregnancy. If the partner of a male subject exposed to the study drug becomes pregnant during the clinical study, the subject may continue participation in the study. The pregnancy must be reported to the sponsor, Henlius, and SciClone's pharmacovigilance departments within 24 hours of the investigator's awareness of the pregnancy.

The investigator shall continuously monitor subjects who become pregnant and follow up on the pregnancy outcomes until 8 weeks after maternal delivery, and report the results to the sponsor, Henlius, and SciClone.

If the pregnancy outcome is stillbirth, spontaneous abortion, fetal malformation (any congenital anomaly/birth defect), or medically indicated termination of pregnancy, it shall be considered an SAE and must be reported following the procedures and within the timelines for SAEs.

If a subject experiences an SAE during pregnancy, it shall be reported following the SAE reporting procedures.

- 1. **Immune-Related Adverse Events**

Given that the mechanism of action of Serplulimab involves T-cell activation and proliferation, irAEs may be observed during this study. Subjects should be monitored for signs and symptoms of irAEs. In the absence of a clearly identified alternative etiology (e.g., infection), signs or symptoms of illness occurring in the subject during the study should be considered related to the immune system.

For dose adjustments of Serplulimab and management principles for AEs, please refer to Section 8 of the protocol.

1. **Statistical Approach**
   1. **Statistical Analysis Data Set**

Full Analysis Set (FAS): Based on the principle of ITT, including patients who have received Serplulimab and with one efficacy evaluation. For case data where the full treatment course is not observed, the last observation carried forward (LOCF) will be used to carry forward the last observation data to study final result. ITT is the primary study population for this study.

Per-protocol Set (PPS): Including all patients that comply with the study protocol, have good compliance, do not take prohibited medications during the study, and complete the required content in the case report forms. No imputation will be made for missing data. Statistical analyses of the drug's efficacy will be performed based on both the FAS and PPS.

Safety Analysis Set: Including all enrolled patients who have received at least one complete cycle of neoadjuvant immunotherapy plus chemotherapy and have safety records after using Serplulimab. This data set is used for safety analysis.

- 1. **Statistical Analysis Plan**

The results of this study will be primarily analyzed using statistical descriptive methods. For measurement data, the mean, standard deviation, median, max, and min values will be listed. For enumeration data and ranked data, the frequency (constituent ratio), rate, and CIs will be listed.

All statistical analyses will be programmed and calculated using SAS 9.2 Statistical Analysis Software. All statistical tests will be performed using two-sided tests, where P < 0.05 will be considered statistically significant for the tested differences, and CIs will be presented as 95% CIs.

- 1. **Efficacy Analysis**

The primary efficacy endpoint is the pCR rate. The ORR and its 95% CI will be calculated by the best overall response (BOR).

The Kaplan-Meier method will be used to plot the DFS curve and estimate the median DFS with its 95% CI, as well as the OS curve and its 95% CI.

Statistical analysis will be performed using SAS 9.4 software, where P < 0.05 will be considered statistically significant for the tested difference.

- 1. **Drug Safety Evaluation**

The AEs and adverse reactions that occurred in this study will be primarily described by descriptive statistical analysis, with a listing provided (adverse reactions are defined as “AEs with a ‘definitely related/probably related/possibly related’ relationship to the study drug”). The laboratory test results describe the conditions where values are normal before treatment but become abnormal afterward, along with the relationship with the study drug in case of abnormal changes. Chi-square test will be performed for the incidence of related adverse reactions between different stratified groups.

1. **Quality Control and Quality Assurance**

The study personnel must be physicians trained in clinical trials and work under the supervision of senior professionals.

The clinical wards are inspected before the study, which must meet standardized requirements to ensure the availability of complete resuscitation equipment.

It is recommended that medications be administered to patients by professional healthcare personnel who have a thorough understanding of the drug regimen to ensure patient compliance.

The investigator must accurately complete the case report form.

The investigator shall adhere to standard operating procedures, oversee the conduct of the clinical study, verify that all data are recorded and reported accurately and completely, and all case report forms are completed correctly and are consistent with source documents, and guarantee that the study is conducted in compliance with the clinical study protocol.

In the event of an SAE, it must be promptly reported to the Ethics Committee, and the study may be temporarily suspended if necessary.

1. **Ethical, Regulatory, and Administrative Principles**
   1. **Ethical Principles**

This study will be conducted in accordance with the principles established by the 18th World Medical Association (Helsinki, 1964) and all subsequent amendments.

- 1. **Laws and Regulations**

This study will be conducted in compliance with all applicable laws and regulations.

- 1. **Data Protection**

Patient personal data and investigator personal data that may be included in the EDC system shall be processed in accordance with all applicable local laws and regulations.

When personal data related to investigators and/or patients are archived or processed, the study director or principal investigator shall take all appropriate measures to protect and prevent unauthorized third parties from accessing such data.

- 1. **Confidentiality Agreement**

All materials, information (oral or written), and unpublished documents provided to the investigator (or any actions performed by the sponsor on behalf of the investigator), including this protocol and CRF, shall not be disclosed by the investigator or study personnel at each site to unauthorized personnel without the prior formal written consent of the leading site.

Except for information that may be disclosed as permitted by regulations, the investigator shall maintain confidentiality regarding all information received, obtained, or derived during this study and shall take all necessary steps to ensure no unauthorized disclosure occurs.

- 1. **Record Safekeeping**

The investigator shall arrange for the safekeeping of study documents until the completion of the study. In addition, for the safekeeping of patient records, the investigator must comply with specific local regulations/guidelines.

Unless otherwise specified in the investigator agreement, it is recommended that the investigator retains study documents for at least five years after the completion or termination of the study, in accordance with other standards and/or local regulations.

- 1. **Early Suspension of Study**

The sponsor may decide to suspend this study at any time and for any reason; the decision to suspend the study will be communicated in writing to the investigators at each site.

Similarly, if the investigator at each site decide to withdraw from the study, he/she must notify the sponsor in writing.

Where applicable, the Institutional Review Board (IRB) and health regulatory authorities should be notified in accordance with local regulations.

- 1. **Sponsor's Audits and Regulatory Authority's Inspections**

The investigators at each site agrees to allow the sponsor's auditors/inspectors from regulatory authorities have direct access to patient study records for review purposes, and acknowledge that these individuals are bound by professional confidentiality principles, thus they will not disclose any personally identifiable or personal medical information of the patients.

The investigator at each site will make every effort to facilitate audits and inspections, ensuring that auditors/inspectors have access to all necessary equipment, data, and documents.

During these inspections, the confidentiality of validation data and the protection of patients shall be respected.

The investigators at each site should promptly communicate the results and information provided by regulatory authorities following inspections to the sponsor.

The investigators at each site shall take appropriate measures as required by the sponsor to implement corrective actions for all issues identified during audits or inspections.

- 1. **Protocol Amendments**

Any amendments to the protocol will be documented in a written revision, and signed by the investigators at each site. The signed revision will be appended to this protocol.

Amendments to this protocol may be submitted in accordance with local regulations.

- 1. **Ownership and Use of Study Data and Results**

The sponsor has full access to the final data to enable appropriate academic analysis and reporting of the study results.

- 1. **Publication**

Any subsequent disclosure or publication by study participants (including substudies) must be approved by the sponsor and cite this study and the initial publication.

The sponsor may request that the sponsor's name and/or the name(s) of one or more employees of the sponsor be included or excluded from this publication.

The sponsor may delay publication or communication for a limited period to protect the confidentiality or proprietary nature of any information contained therein.
