# Supplementary material for: Neoadjuvant immunochemotherapy plus thymalfasin in locally advanced gastric cancer: a prospective clinical trial
Source: BMC Med. 2026 Feb 26;24:143. doi: 10.1186/s12916-026-04740-z (PMC12964648; doi:10.1186/s12916-026-04740-z)
Supplement: Supplementary file 2 — Additional file 2: Table S1. Perioperative and surgical outcomes. [file 12916_2026_4740_MOESM2_ESM.docx]

**Table S1.** Perioperative and surgical outcomes.

| **Outcomes, *n* (%)** | **Patients (*n* = 30)** |
| --- | --- |
| **Surgical Approach** |  |
| Robotic gastrectomy | 6 (20.0) |
| Total laparoscopic gastrectomy | 15 (50.0) |
| Laparoscopic-assisted gastrectomy | 9 (30.0) |
| **Surgery pattern** |  |
| Total gastrectomy | 15 (50.0) |
| Distal gastrectomy | 13 (43.3) |
| Proximal gastrectomy | 2 (6.7) |
| **Operative time (min)** | 206 (177-226) |
| **Intraoperative blood loss (mL)** | 50 (30-65) |
| **Postoperative hospital stays (days)** | 7 (5-8) |
| **Perioperative complications** |  |
| Pulmonary infection, grade II | 1 (3.3) |
| Pleural effusion, grade II | 1 (3.3) |
| Gastrointestinal fistula, grade IIIb | 1 (3.3) |

Note: Continuous variables are summarized as median with interquartile range (IQR).
