# Supplementary material for: Neoadjuvant immunochemotherapy plus thymalfasin in locally advanced gastric cancer: a prospective clinical trial
Source: BMC Med. 2026 Feb 26;24:143. doi: 10.1186/s12916-026-04740-z (PMC12964648; doi:10.1186/s12916-026-04740-z)
Supplement: Supplementary file 3 — Additional file 3: Table S2. Baseline characteristics and comparability of the study cohorts. [file 12916_2026_4740_MOESM3_ESM.docx]

**Table S2.** Baseline characteristics and comparability of the study cohorts.

| **Characteristics** | **GATES IIT Cohort**  **(*n* = 30)** | **GATES RNA-seq Subset**  **(*n* = 20)** | ***P1* value** | **Control RNA-seq Cohort**  **(*n* = 16)** | ***P2* value** |
| --- | --- | --- | --- | --- | --- |
| **Age, years** |  |  | 0.494 |  | 0.182 |
| ≤ 65 | 15 (50.0) | 8 (40.0) |  | 10 (62.5) |  |
| > 65 | 15 (50.0) | 12 (60.0) |  | 6 (37.5) |  |
| Sex |  |  | 1.000 |  | 0.380 |
| Female | 3 (10.0) | 2 (10.0) |  | 4 (25.0) |  |
| Male | 27 (90.0) | 18 (90.0) |  | 12 (75.0) |  |
| Smoking |  |  | 0.734 |  | 0.658 |
| No | 15 (50.0) | 11 (55.0) |  | 10 (62.5) |  |
| Yes | 15 (50.0) | 9 (45.0) |  | 6 (37.5) |  |
| Drinking |  |  | 0.632 |  | 0.343 |
| No | 20 (66.7) | 12 (60.0) |  | 12 (75.0) |  |
| Yes | 10 (33.3) | 8 (40.0) |  | 4 (25.0) |  |
| Tumor location |  |  | 0.810 |  | 0.628 |
| Gastric cancer | 20 (66.7) | 14 (70.0) |  | 10 (62.5) |  |
| EGJ cancer | 10 (33.3) | 6 (30.0) |  | 6 (37.5) |  |
| H.P infection |  |  | 1.000 |  | 1.000 |
| No | 9 (30.0) | 6 (30.0) |  | 5 (31.3) |  |
| Yes | 21 (70.0) | 14 (70.0) |  | 11 (68.8) |  |
| Tumor diameter, cm |  |  | 1.000 |  | 1.000 |
| ≤ 5.0 | 15 (50.0) | 10 (50.0) |  | 8 (50.0) |  |
| > 5.0 | 15 (50.0) | 10 (50.0) |  | 8 (50.0) |  |
| Differentiation grade |  |  | 0.908 |  | 0.641 |
| G2 | 13 (43.3) | 9 (45.0) |  | 6 (37.5) |  |
| G3 | 17 (56.7) | 11 (55.0) |  | 10 (62.5) |  |
| Lauren classification |  |  | 0.969 |  | 0.465 |
| Diffused | 10 (33.3) | 7 (35.0) |  | 4 (25.0) |  |
| Intestinal | 16 (53.3) | 10 (50.0) |  | 7 (43.8) |  |
| Mixed | 4 (13.3) | 3 (15.0) |  | 5 (31.3) |  |
| HER2 status |  |  | 0.733 |  | 0.221 |
| - | 18 (60.0) | 11 (55.0) |  | 12 (75.0) |  |
| 1+ | 12 (40.0) | 9 (45.0) |  | 4 (25.0) |  |
| DNA mismatch repair |  |  | 1.000 |  | 1.000 |
| pMMR | 28 (93.3) | 19 (95.0) |  | 16 (100) |  |
| dMMR | 2 (6.7) | 1 (5.0) |  | 0 (0) |  |
| PD-L1 status |  |  | 0.770 |  | 0.076 |
| CPS <1 | 11 (36.7) | 9 (45.0) |  | 5 (31.3) |  |
| CPS 1-5 | 7 (23.3) | 4 (20.0) |  | 9 (56.3) |  |
| CPS ≥5 | 12 (40.0) | 7 (35.0) |  | 2 (12.5) |  |
| Pretreated cT stage |  |  | 1.000 |  | 0.453 |
| T3 | 15 (50.0) | 10 (50.0) |  | 6 (37.5) |  |
| T4 | 15 (50.0) | 10 (50.0) |  | 10 (62.5) |  |
| Pretreated cN stage |  |  | 0.654 |  | 1.000 |
| N2 | 17 (56.7) | 10 (50.0) |  | 8 (50.0) |  |
| N3 | 13 (43.3) | 10 (50.0) |  | 8 (50.0) |  |
| Surgery pattern |  |  | 1.000 |  | 1.000 |
| Total gastrectomy | 15 (50.0) | 10 (50.0) |  | 8 (50.0) |  |
| Partial gastrectomy | 15 (50.0) | 10 (50.0) |  | 8 (50.0) |  |
| Pathological T stage |  |  | 0.580 |  | 0.337 |
| ypT0 | 9 (30.0) | 8 (40.0) |  | 5 (31.3) |  |
| ypT1 | 1 (3.3) | 1 (5.0) |  | 1 (6.3) |  |
| ypT2 | 4 (13.3) | 4 (20.0) |  | 2 (12.5) |  |
| ypT3 | 10 (33.3) | 3 (15.0) |  | 5 (31.3) |  |
| ypT4 | 6 (20.0) | 4 (20.0) |  | 3 (18.8) |  |
| Pathological N stage |  |  | 0.285 |  | 0.009* |
| ypN0 | 19 (63.3) | 16 (80.0) |  | 6 (37.5) |  |
| ypN1 | 5 (16.7) | 2 (10.0) |  | 2 (12.5) |  |
| ypN2 | 1 (3.3) | 1 (5.0) |  | 5 (31.3) |  |
| ypN3 | 5 (16.7) | 1 (5.0) |  | 3 (18.8) |  |
| Downstaging of T stage |  |  | 0.535 |  | 0.678 |
| No | 10 (33.3) | 5 (25.0) |  | 5 (31.3) |  |
| Yes | 20 (66.7) | 15 (75.0) |  | 11 (68.8) |  |
| Downstaging of N stage |  |  | 0.450 |  | 0.380 |
| No | 6 (20.0) | 2 (10.0) |  | 4 (25.0) |  |
| Yes | 24 (80.0) | 18 (90.0) |  | 12 (75.0) |  |
| Downstaging of TNM stage |  |  | 0.501 |  | 0.143 |
| No | 7 (23.3) | 3 (15.0) |  | 6 (37.5) |  |
| Yes | 23 (76.7) | 17 (85.0) |  | 10 (62.5) |  |
| Major pathological response (MPR) |  |  | 0.564 |  | 0.195 |
| No | 13 (43.3) | 7 (35.0) |  | 9 (56.3) |  |
| Yes | 17 (56.7) | 13 (65.0) |  | 7 (43.8) |  |

Note: P1 values were derived from comparisons between the GATES ITT cohort (n=30) and the GATES RNA-seq subset (n=20). P2 values resulted from comparisons between the GATES RNA-seq subset (n=20) and the external Control RNA-seq cohort (n=16). Both used Fisher's exact test for categorical variables. * P<0.05.
